# Atypical DNA methylation, sRNA-size distribution, and female gametogenesis in *Utricularia gibba*

**DOI:** 10.1038/s41598-021-95054-y

**Published:** 2021-08-03

**Authors:** Sergio Alan Cervantes-Pérez, Lenin Yong-Villalobos, Nathalia M. V. Florez-Zapata, Araceli Oropeza-Aburto, Félix Rico-Reséndiz, Itzel Amasende-Morales, Tianying Lan, Octavio Martínez, Jean Philippe Vielle-Calzada, Victor A. Albert, Luis Herrera-Estrella

**Affiliations:** 1 Laboratorio Nacional de Genómica para la Biodiversidad, Centro de Investigación y de Estudios Avanzados del Instituto Politécnico Nacional, 36824 Irapuato, Guanajuato Mexico; 2 Department of Biological Sciences, University at Buffalo, Buffalo, NY 14260 USA; 3 School of Biological Sciences, Nanyang Technological University, Singapore, 637551 Singapore; 4 Present Address: Institute of Genomics for Crop Abiotic Stress Tolerance, Plant and Soil Department, Texas Tech University, Lubbock, TX 79409 USA; 5 Present Address: Instituto de Investigación de Recursos Biológicos Alexander von Humboldt, Avenida Paseo Bolívar (Circunvalar) #16-20, Bogotá, DC 111311 Colombia

**Keywords:** Computational biology and bioinformatics, Genome informatics

## Abstract

The most studied DNA methylation pathway in plants is the RNA Directed DNA Methylation (RdDM), a conserved mechanism that involves the role of noncoding RNAs to control the expansion of the noncoding genome. Genome-wide DNA methylation levels have been reported to correlate with genome size. However, little is known about the catalog of noncoding RNAs and the impact on DNA methylation in small plant genomes with reduced noncoding regions. Because of the small length of intergenic regions in the compact genome of the carnivorous plant *Utricularia gibba,* we investigated its repertoire of noncoding RNA and DNA methylation landscape. Here, we report that, compared to other angiosperms, *U. gibba* has an unusual distribution of small RNAs and reduced global DNA methylation levels. DNA methylation was determined using a novel strategy based on long-read DNA sequencing with the Pacific Bioscience platform and confirmed by whole-genome bisulfite sequencing. Moreover, some key genes involved in the RdDM pathway may not represented by compensatory paralogs or comprise truncated proteins, for example, *U. gibba* DICER-LIKE 3 (DCL3), encoding a DICER endonuclease that produces 24-nt small-interfering RNAs, has lost key domains required for complete function. Our results unveil that a truncated DCL3 correlates with a decreased proportion of 24-nt small-interfering RNAs, low DNA methylation levels, and developmental abnormalities during female gametogenesis in *U. gibba*. Alterations in female gametogenesis are reminiscent of RdDM mutant phenotypes in *Arabidopsis thaliana*. It would be interesting to further study the biological implications of the DCL3 truncation in *U. gibba*, as it could represent an initial step in the evolution of RdDM pathway in compact genomes.

## Introduction

DNA modifications are chemical additions to DNA and/or histones that are associated with changes in gene expression and can be heritable^1^. One of these DNA modifications is the methylation at the 5ʹ position of the cytosine base (5mC), an ancient evolutionary trait associated with gene and transposable element (TE) silencing in eukaryotes^2^. In plant genomes, 5mC is a widely conserved DNA modification that modulates gene expression and plays a key role in many developmental processes and environmental responses^3,4^.

Non-coding RNAs (ncRNAs) are fundamental in regulating DNA methylation and serving as scaffolds for heterochromatin formation and silencing of genes^5^. For instance, mobile short RNAs (sRNAs) underlie shoot to root communication of methylation status^6^ and long-noncoding RNAs (lncRNAs) serve as scaffolds in *de novo* DNA methylation^7^. RNA-directed DNA methylation (RdDM) is the major small RNA-mediated DNA methylation pathway in plants. The canonical pathway can be subdivided into 3 different phases: (1) RNA polymerase IV (Pol-IV) dependent biogenesis of small interfering RNAs (siRNAs), (2) RNA polymerase V (Pol-V) mediated *de novo* methylation, and (3) chromatin modifications^8^. In addition to Pol-IV and Pol-V, other key components in RdDM and other sRNA biogenesis pathways key protein families are the ARGONAUTE (AGO), DICER-LIKE (DCL), and RNA DEPENDENT RNA POLYMERASE (RDR). Specifically, in the production of siRNAs, RDRs synthesize the second RNA strand, DCLs process RNA precursors, and AGOs select one DNA strand and load sRNAs to a specific target^8,9^.

A considerable number of plant methylomes have been analyzed by whole-genome bisulfite sequencing (BS-Seq) or by high-performance liquid chromatography^10,11^, where the vast majority of these methylomes are from model flowering plants. Methylome analysis has revealed variation of methylation patterns in intergenic and gene body regions among different plant species^11,12^ and these patterns could be related to genome architectural features such as genome size, rearrangements, duplications, and content/expansion of transposable elements (TEs), among others. Little is known about the relationship between DNA methylation, number, type of ncRNA *loci*, and genome size, particularly for plant species with small genomes with reduced TEs and other repetitive sequences.

*Utricularia gibba* is an aquatic, carnivorous plant belonging to the asterid lineage that despite having undergone two recent whole genome duplication (WGD) events, has a remarkably small genome size (ca. 100 Mb)^13^. *U. gibba* is a rootless plant that harbors slender green stems that grow like stolons with alternate thread-like leaves and numerous complex trapping bladders that catch small invertebrate prey. *U. gibba* has a gene repertoire similar to other plants species with larger genomes, albeit with a reduced non-coding genome harboring short intergenic regions (IGR) and a low TE content^13^. Here, we took advantage of the small *U. gibba* genome to study the impact of genome size on the conservation of “non-coding DNA”, especially on nature of genes coding for ncRNAs and the impact of the ncRNA landscape on DNA methylation. Here, we report that *U. gibba* has, compared to Arabidopsis, a functionally incomplete canonical pathway for siRNAs biogenesis and *de novo* methylation that correlates with a rather unusual content and proportion of sRNA. In addition, our data suggests the interesting hypothesis that the unusual siRNA content and altered female gametogenesis in *U. gibba* could be due to the lack of a functional *DCL3*. Finally, we suggest that Pacific Bioscience (PacBio) Single Molecule Real-Time (SMRT) long read sequencing data to determine 5mC methylomes is a useful alternative to BS-seq.

## Results

### *U. gibba* lncRNAs has a reduced number of intergenic lncRNAs

**Figure 1:**
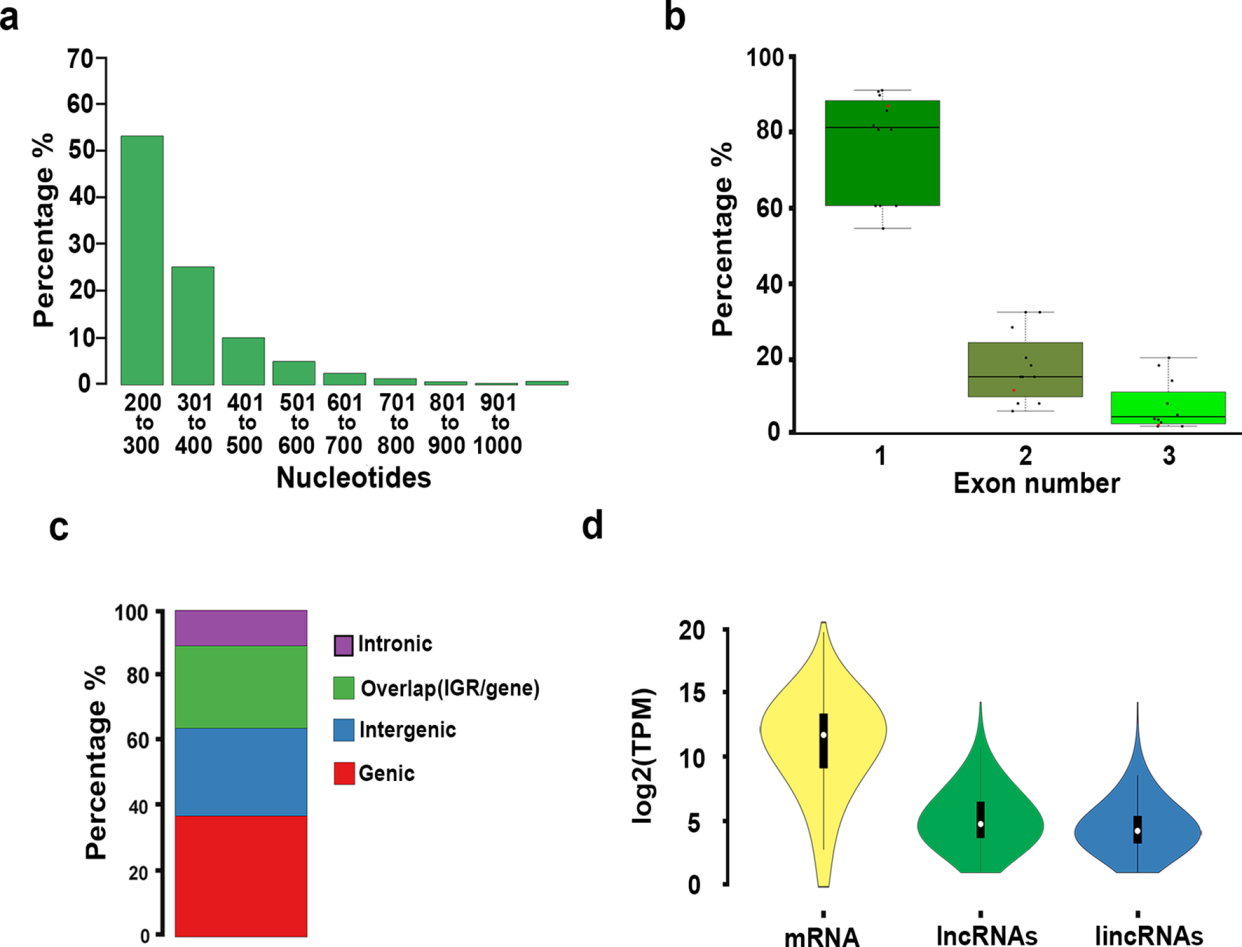
Architecture and annotation of lncRNAs in *U. gibba* and other plants. (**a**) lncRNAs size distribution in *U. gibba* is shown in a barplot grouping each 100-nt in size (X axis) and by percentage of the total (Y axis). (**b**) Structure of lncRNAs in selected plants (Additional file 1: Dataset S1). The box plot groups the proportion of exon number (X axis) by percentage (Y axis) among lncRNAs identified from various plant genomes. (**c**) Genomic annotation of *U. gibba* lncRNAs. The proportion is from 4295 putative lncRNAs loci identified. Red represents the proportion of lncRNAs mapped to body gene regions; blue shows lncRNAs located in intergenic regions; green indicates those that overlap gene body and intergenic regions. (**d**) Coding and noncoding transcripts expression. The violin plot represents the total of mRNAs, lncRNAs and lincRNAs identified in this study with correspondent log2(tpm).

**Figure 2:**
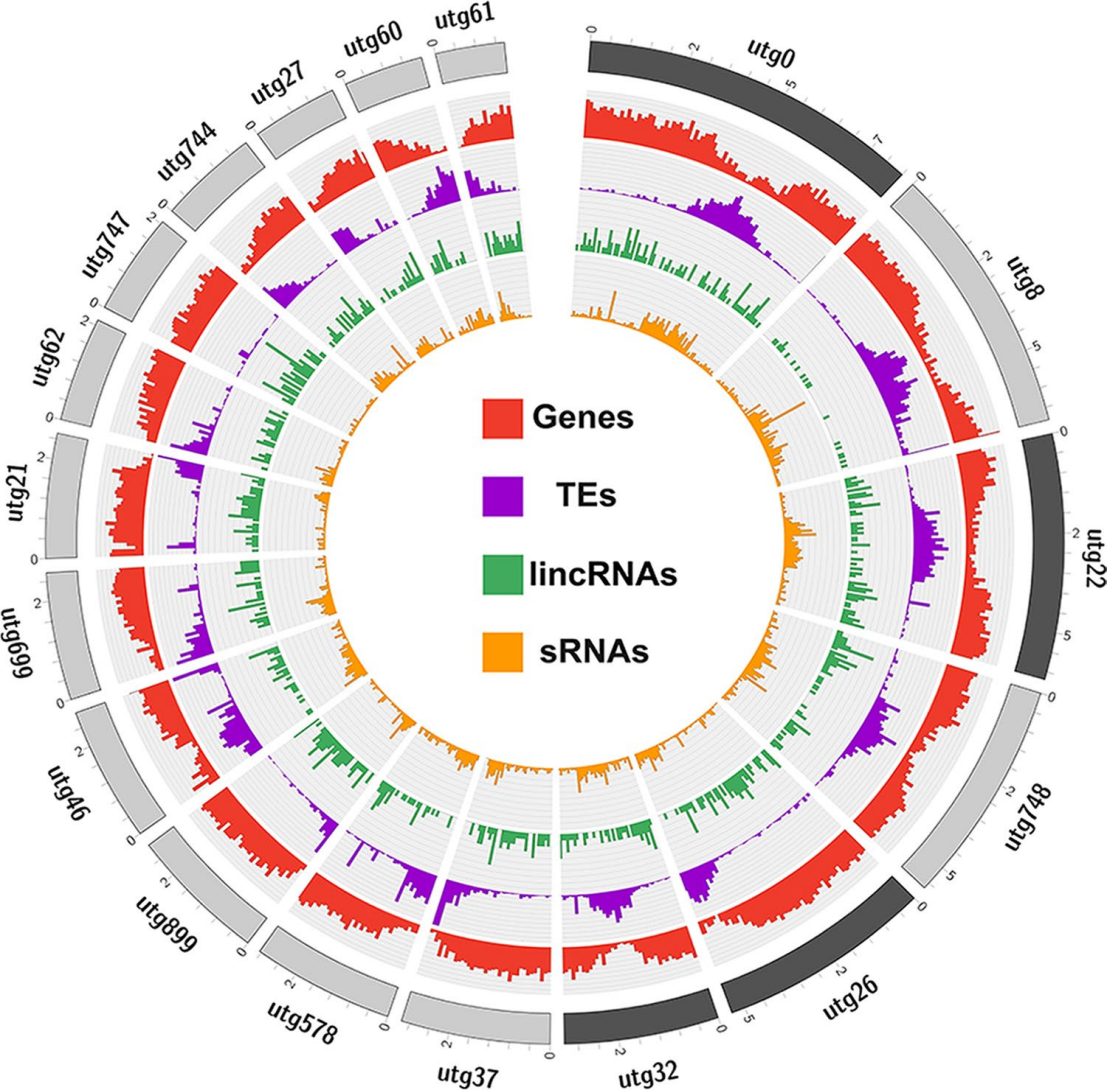
Noncoding RNA landscape in *Utricularia gibba.* Representation of the *U. gibba* genome in a circus plot where the outside blocks represent complete chromosomes (dark gray) and Unitigs or contigs (gray) larger than 1 Mb in size. The density plots were calculated in 10Kb windows. Red histogram shows the gene density. The Purple histogram displays transposable element density. lincRNA density is represented in the green histogram, and small RNA density for sRNAs 20-nt to 24-nt in size are shown in orange.

lncRNAs were obtained from sequencing data of 12 RNA-Seq libraries, 10 from vegetative and 2 from trap tissue from plants subjected to the contrasting abiotic stress or hormone treatments (Additional file 1: Dataset S1). We produced over 19 million mapped reads per library (95.37% mapping to the genome) for a total of 330,258,977 million mapped reads (Additional file 1: Dataset S1). To identify lncRNAs, assembled transcripts were translated into the three potential reading frames and filtered by homology to proteins encoded in the *U. gibba* genome and other plants in a non-redundant protein database^14^, yielding 10,386 putative lncRNAs of at least 200-nt in length. Putative lncRNAs were then filtered to eliminate precursors of tRNAs, rRNAs, miRNAs among others. Putative lncRNAs were further filtered for coding potential in sense-direction, resulting in 4,295 putative lncRNAs including possible antisense, intronic, and intergenics (Additional file 2: Fig. S1). The vast majority of these lncRNA *loci* were relatively short, 89.05% were smaller than 500-nt, and only 1.81% longer than 800-nt (Fig. 1a). The lncRNA mean length was 336.9-nt and the largest one was 2134-nt (Additional file 2: Table S1; Additional file 3: Dataset S2). We also found that in *U. gibba* 86.51% of lncRNAs had a single exon structure and 13.49% had two or more exons, percentages like other plant species for which lncRNAs have been reported (Fig. 1b; Additional file 1: Dataset S1). When putative lncRNAs were mapped onto the *U. gibba* genome, we determined that 37.03% mapped to regions corresponding to exons of protein coding genes in antisense orientation, 10.89% to intronic regions, 25.26% overlapped gene bodies and IGRs, and 26.82% were located only in IGRs (Fig. 1c). As it is more complex to determine whether a non-coding transcript that overlaps with an mRNA indeed corresponds to a lncRNA, we only made comparison of the U. gibba intergenic lncRNAs (lincRNAs) with other plant species. We found that this carnivorous plant, under all conditions tested, expresses a total of 1152 lincRNAs, which is lower than that reported for other plant species that ranges between 1580 and 3100 lincRNAs (Additional file 2: Fig. S2). Additionally, were evaluated the expression profiles for coding and noncoding transcripts and we found a higher level of expression for mRNA than for the entire catalog of lncRNAs or lincRNAs (Fig. 1d). To visualize the distribution lincRNAs *loci*, reads were mapped onto the 18 largest contigs of the *U. gibba* genome (>1Mb), which included 4 putatively entire chromosomes. *loci* encoding lncRNAs were distributed across the entire genome with prevalence in high gene density regions, and low frequency in regions that are flanking centromere and contain repetitive sequences and transposable elements, named pericentromeric regions, which can be more clearly seen in the four complete chromosomes (Fig. 2; dark grey color). lncRNAs density was similar in 17 of the largest contigs of the *U. gibba* genome^15^, except in Unitig_8 (6.8 Mb) that has considerably lower density of lncRNA *loci* (Fig. 2).

### *U. gibba* has an atypical abundance of 24-nt sRNAs compared to other angiosperms

To further characterize noncoding RNA diversity in *U. gibba*, we carried out small RNA-Seq analysis of RNAs extracted from green tissue and traps from plants grown under the same conditions described for lncRNA identification. We obtained a total of 23.6 million mapped reads, of which 19.9 million had lengths between 20 to 25-nt, which were selected for further analyses (Additional file 1: Dataset S1). Upon mapping the reads onto the *U. gibba* genome, we found that *loci* for 20 to 25-nt sRNAs are mainly located at pericentromeric regions, where TE density is higher, but with some peaks in regions of high gene content (Fig. 2). Similar results have been published previously for other plant species^16^.

**Figure 3:**
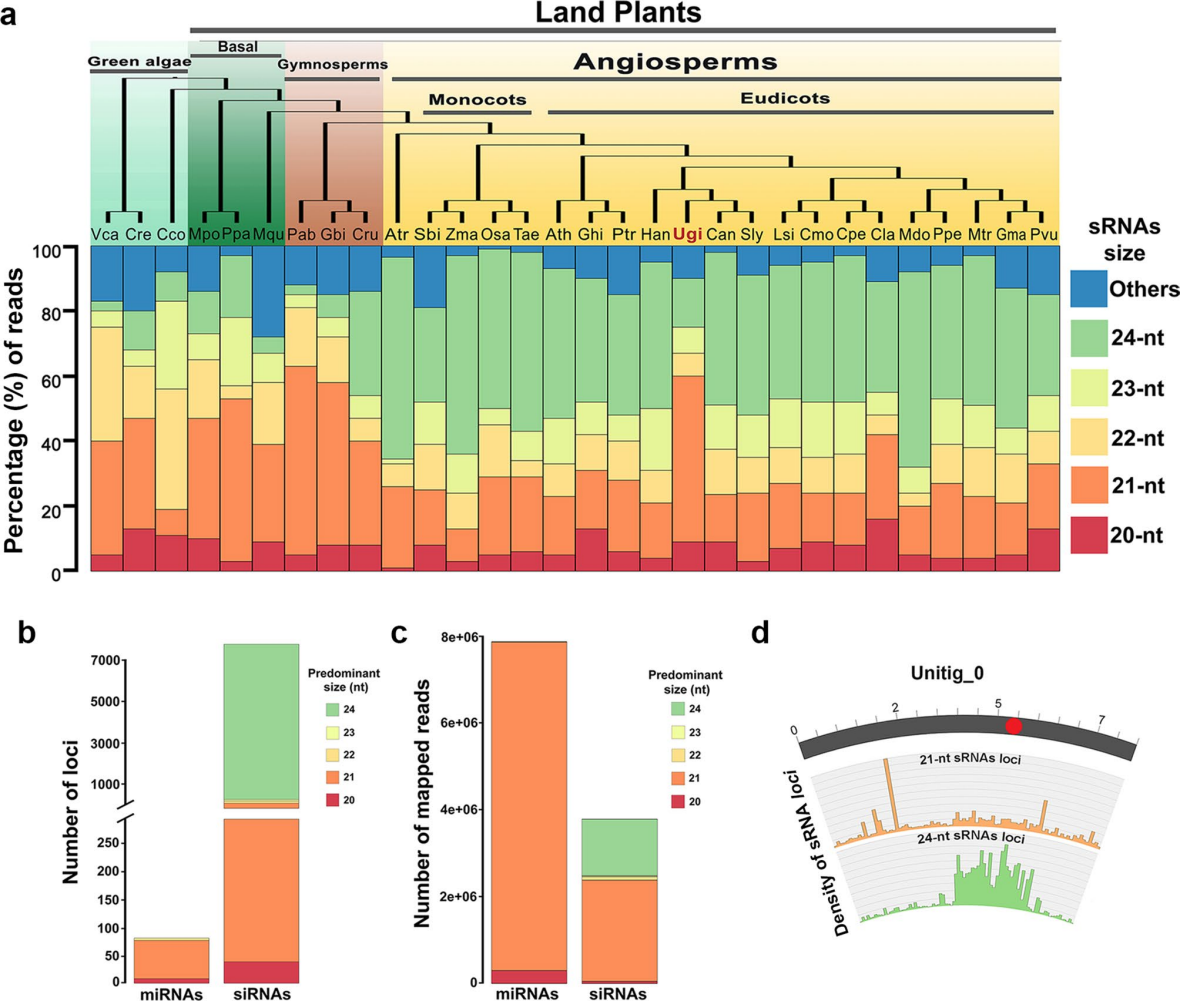
Annotation of sRNAs in *U. gibba* and distribution of small RNA size abundance in plants. (**a**) Top, a phylogenetic tree of plants with representative species for green algae, early branching land plants, gymnosperms, and angiosperms. Bottom, a stacked plot of small RNA abundances (20-nt to 24-nt in size) from various small RNA-seq studies. Y-axis shown the percentage of reads for each size group. (**b**) Total locus predictions from ShortStack for miRNAs and siRNAs at different sizes. (**c**) Numbers of reads mapped per loci at different sizes from ShortStack. (**d**) The largest complete chromosome (Unitig_0) representation of gene density, TE density, 21-nt small RNAs loci density distribution and 24-nt small RNAs loci distribution. *Vca: Volvox carteri; Cre: Chlamydomonas reinhardtii; Cco: Chara coraline; Mpo: Marchantia polymorpha; Ppa: Physcomitrella patens; Mqu: Marsilea quadrifolia; Pab: Picea abies; Ginkgo biloba; Cru: Cycas rumphii; Atr: Amborella trichopoda; Sbi: Sorghum bicolor; Zma: Zea mays; Osa: Oryza sativa; Tae: Triticum aestivum; Ath: Arabidopsis thaliana; Ghi: Gossypium hirsutum; Ptr: Populus trichocarpa; Han: Helianthus annuus; Ugi: Utricularia gibba; Can: Capsicum annuum; Sly: Solanum lycopersicum; Lsi: Lagnaria siceraria; Cmo: Cucurbita moschata; Cpe: Cucurbita pepo; Cla: Citrullus lanatus; Mdo: Malus domestica; Ppe: Prunus persica; Mtr: Medicago truncatula; Gma: Glycine max; Pvu: Phaseolus vulgaris*.

An interesting finding was that most sRNA sequencing reads corresponded to 21-nt sRNAs (52.8%) and only 14.4% to 24-nt sRNAs (Additional file 1: Dataset S1). This contrasts with sRNA size distribution for other angiosperms, for which the most abundant sRNA class is 24-nt^17,18^. To confirm that the sRNA size distribution in *U. gibba* differs from that of other angiosperms, we performed an analysis of sRNA size abundance in representative plants from different clades for which sRNAs have been characterized (Additional file 1: Dataset S1). We analyzed sRNA datasets for total reads for 30 plant species, of which 21 were angiosperms and 9 were representative plants outside the angiosperms. Our results confirm that in both monocot and eudicot species, except for *U. gibba*, the most abundant sRNA class is 24-nt (Fig. 3a). For the case of green algae, the most prevalent sRNAs classes are of 21 to 23-nt, whereas in *Volvox carteri* the most abundant sRNAs are of 21-nt and 22-nt, in *Chara coraline* the most abundant are 22-nt and 23-nt (about 30% of each size), and for *Chlamydomonas reinhardtii* the 21-nt class predominates (Fig. 3a). In early-branching land plants (*Marchantia polymorpha, Physcomitrella patens*, and *Marsilea quadrifolia*) and gymnosperms (*Picea abies*, *Gynkgo biloba*) the most abundant size class of sRNAs is 21-nt, except for *Cycas rumphii*, which has similar amounts of 21 and 24-nt sRNAs (Fig. 3a). However, the distribution of sRNAs could vary among tissues, therefore, it will be interesting in the future to have information about the tissue-specific or even cell type-specific distribution of sRNAs to provide a more specific landscape of sRNAs in plants.

### A large number of 24-nucleotide small RNA *loci* produce a small proportion of sRNA reads and are associated preferentially with intergenic regions in *U. gibba*

In general, sRNA sequence distribution in angiosperms is characterized by a major 24-nt peak containing primarily unique reads, and a 21-nt peak comprising many redundant reads^19^. As expected this is also true in model angiosperm species such as Arabidopsis^20^, tomato^21^ and rice^22^. For the three samples we sequenced (Additional file 2: Fig. S3), 21-nt sRNAs had higher redundancy (up to 97% of the reads are redundant) in comparison with 24-nt sRNAs, of which 30% were unique and 70% redundant (Additional file 1: Dataset S1). To better classify sRNA *loci*, we performed an analysis with ShortStack V2.0^23^ to identify *in silico* which *DICER*-like (*DCL*) genes are involved in the biogenesis of miRNAs or siRNAs. ShortStack analysis identified 7478 siRNA *loci* and only 80 miRNA *loci* (Fig. 3b; Additional file 4: Dataset S3), which produce nearly 1.5 million and 8 million mapped reads respectively, suggesting that a large number of siRNA loci accumulate a low number of reads (Fig. 3c). Of the 71 miRNA *loci* identified (Additional file 2: Table S2), 69 already were annotated in the miRBase V22.0 catalog of eukaryotic miRNAs, and 2 represent putative Utricularia specific miRNAs (Additional file 4: Dataset S3; Additional file 2: Fig. S4). miRNA *loci* grouped into 17 families, of which the miR166, miR156, miR159, miR319 and miR858 families made up 94% of the miRNA reads (Additional file 2: Fig. S5). These miRNA families are conserved in most plant species and produce high levels of mature miRNAs^18^.

sRNA *loci* annotation (excluding TE annotation) reveals that the 24-nt sRNA *loci* are preferentially located at IGRs with 80% of them, while the sRNA *loci* of 20-nt to 22-nt were located in similar proportions at genic and intergenic regions and for the 23-nt sRNA class, 57% of *loci* are located at IGRs and 31% in genic regions (Additional file 2: Fig. S6).The annotation is consistent with the distribution of sRNAs at the genome scale and can be clearly seen in unitig_0, in which 21-nt sRNA *loci* are distributed across the chromosome with significant peaks in regions with high gene density and 24-nt sRNA *loci* were found to be predominantly located in regions with high TE density, presumably pericentromeric regions (Fig. 3d).

### sRNA biogenesis and the RdDM pathway in *U. gibba*

The unusual proportions of 24-nt and 21-nt sRNAs observed in *U. gibba* suggest that its sRNA production machinery could differ somehow from those of other angiosperms. To explore this possibility, we focused on the presence of genes involved canonical miRNA biogenesis, genes that are part of the subunits of RNA Pol-IV and RNA Pol-V, homologs of AGO, DCL, RDR, and other key genes involved in siRNA biogenesis and *de novo* DNA methylation in *U. gibba*. We searched based on sequence homology (transcript and protein), protein domain conservation, synteny, and through phylogenetic analysis. Furthermore, we performed a comparison with representative plant species (both angiosperm and non-angiosperm) for which key RdDM pathway genes^24^ and RNA polymerase compositions were previously reported^25^.

We found that key genes involved in canonical miRNA biogenesis such as *DICER LIKE 1 (DCL1)*, *SERRATE* (*SE*), *HYPONASTIC LEAVES 1* (*HYL1*), *HASTY 1* (*HST1*) and *ARGONAUTE 1* (*AGO1*) are conserved in *U. gibba* (Additional file 2: Table S3). Pol-IV and Pol-V are crucial in the RdDM pathway and are constituted by diverse DNA-DIRECTED RNA POLYMERASES IV AND V (NRPD/NRPE) proteins. Pol-IV and Pol-V differ from RNA polymerase II in their second, fourth, fifth, and seventh subunits (NRPD2, NRPD4, NRPE5, NRPD7, respectively). The *U. gibba* genome encodes *NRPD1, NRPE1 and NRPD2* genes (Additional file 2: Fig. S7), which is consistent with the presence of siRNAs and their strong conservation in the land plant lineage. We also identified NRPD7 in the *U. gibba* genome (Additional file 2: Fig. S7), a subunit previously identified in green eukaryotes except in the algae Chlamydomonas, and NRPD5, found in gymnosperms and angiosperms but not in algae and ferns^25,26^. Only after an extensive search did we find evidence for an NRPD4 ortholog in *U. gibba*. Although this gene is classified as an orphan in Plaza 4.0 (HOM04D168668) for *Arabidopsis thaliana*, our analysis showed that this categorization is likely related to the *Arabidopsis thaliana* homolog being extremely divergent; even the *A. lyrata* ortholog was readily placed into a clear NRPD4 gene family (Additional file 2: Fig. S8). Interestingly, this subunit has been reported only in angiosperms and not in gymnosperms, early land plants or algae^25,26^.

The AGO protein family in Arabidopsis has been subdivided into 4 clades: AGO2/3/7, AGO4/6/8/9, AGO1/10 and AGO5. The number of family members of this protein family ranges from 2 AGO proteins in *C. reinhardtii* to 10 in Arabidopsis and 20 in *Z. mays*^27^. We searched in the Phylome database v4^28^ for possible homologous genes in *U. gibba* and found evidence for at least one AGO for each clade, with the exception of AGO5 for which no homologs were found (Additional file 2: Fig. S9). Two genes were found for AGO clade 2/3/7 (Additional file 2: Fig. S10), two genes represented the AGO 4/6/8/9 clade (Additional file 2: Fig. S11), and 4 homologs grouped in the AGO 1/10 clade (Additional file 2: Fig. S12). Additionally, we performed our own exhaustive phylogenetic analysis with many plant genomes to assign each *U. gibba AGO* gene with more certainty into specific clades. We were able to identify the same 8 AGOs described above, wherein the 2 homologs of clade 2/3/7 apparently are AGO7 copies, the two 4/6/8/9 copies appear closer to AGO4 than AGO6. AGO8/9 are sister genes only in Brassicaceae. There is one *U. gibba* AGO10 and three homologs for AGO1 in the AGO1/10 clade (Additional file 2: Fig. S13).

**Figure 4:**
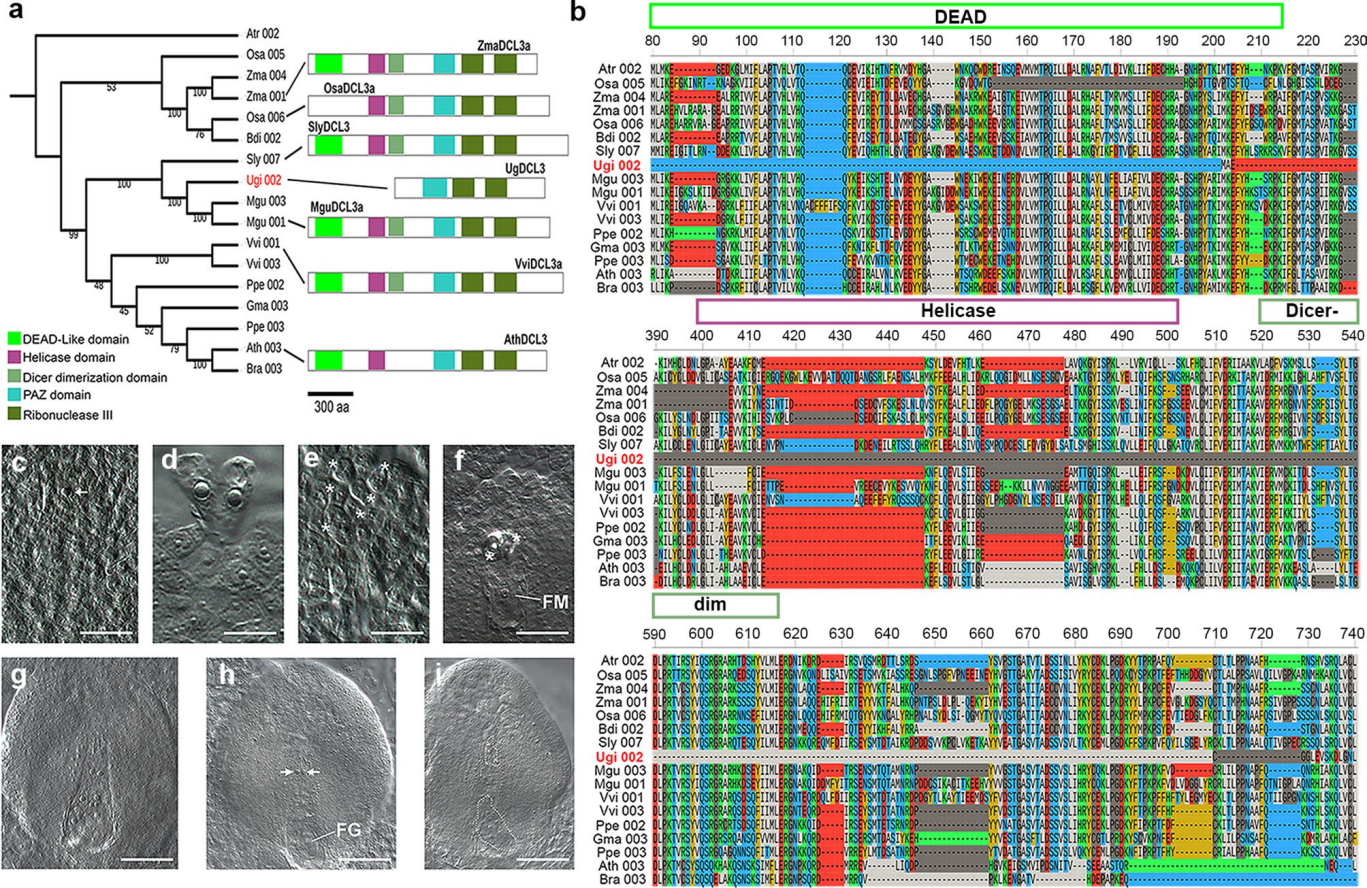
Truncated DCL3 and female gametophyte development in the ovule of *U. gibba.* (**a**) Phylogenetic analysis of DCL3 in some angiosperms including *U. gibba*. A typical DCL3 protein has the DEAD, Helicase, Dicer dimerization, PAZ, and two Ribonuclease III domains, while UgDCL3 only contains the PAZ and Ribonuclease III domains. (**b**) Multiple sequence alignment of DCL3 proteins in blocks of 150 aa showing the missing domains in UgDCL3. (**c**) Developing ovules showing a single pre-meiotic precursor corresponding to the MMC. (**d**) Twin pre-meiotic precursors prior to megasporogenesis. (**e**) Six differentiated cells (asterisk) corresponding to gametic precursors prior to megasporogenesis. (**f**) Functional megaspore (FM) and two degenerated megaspores (asterisk) following megasporogenesis. (**g**) Developing ovule showing a single 8-nuclear female gametophyte. (**h**) Developing ovule showing a female gametophyte (FG) and two ectopic gametic precursors (arrows) at the chalazal region. **i** Developing ovule showing two independent female gametophytes (dashed). Scale bars: 12.5 µm in (**c**) to (**f**); and 20 µm in (**g**) and (**i**).

In seed plants there are three *RDR* ortholog genes; two are conserved in all land plants, *RDR1* and *RDR6,* and *RDR2* is required for production of Pol-IV-siRNAs and is specific to seed plants. Aside from these RDRs, three additional members of this protein family, RDR3, RDR4, and RDR5, are present in Arabidopsis and other plants^29–31^. We found RDR6 (two copies), RDR1 and RDR2, but no evidence for the presence of RDR3/4/5 in the genome of *U. gibba* (Additional file 2: Fig. S14a). In angiosperms the DCL family has 4 members: DCL1, DCL2, DCL3, and DCL4^32^; however, in lycophytes and ferns there is only evidence for the presence of DCL1, DCL3 and DCL4^26^. Phylogenetic analysis of the DCL family permitted the identification in *U. gibba* of 4 DCL proteins (DCL1, DCL2, DCL3, DCL4)*,* suggesting that has a DCL repertoire similar to other angiosperms (Additional file 2: Fig. S14b). Globally, were able to assign *U. gibba* homologs for the remaining key genes in the canonical RdDM pathway (Additional file 1: Dataset S1). However, although UgDCL3 phylogenetically groups very closely to tomato and Mimulus DCL3, UgDCL3 is missing its N-terminal region, where the conserved DEAD/DEAH, Helicase and Dicer dimerization domains are located (Fig. 4a; Additional file 2: Fig. S15). The absence of about 600 amino acids of the N-terminal region in UgDCL3 is evident in a multiple sequence alignment of DCL3 with other angiosperms (Fig. 4b; Additional file 2: Fig. S16). To confirm that the incomplete DCL3 does not represents mistake in the assemble/annotation of the *U. gibba* genome, we searched for the presence of DCL3 transcripts in the different RNA-Seq libraries and we confirm with a 5´ Rapid Amplification of cDNA Ends (5´RACE) that in both cases the sequence of transcripts and 5´ RACE sequence that the *U. gibba* DCL3 gene is missing the DEAD/DEAH domain (Additional file 2: Fig. S17).

### Female gametogenesis in *U. gibba* is reminiscent of Arabidopsis mutants affected in the RdDM pathway

Mutations affecting most of the genes involved in the RdDM pathway have no obvious phenotype during the vegetative development of plants, but show defects in female gametogenesis, including the differentiation of supernumerary gametic precursors that often give rise to ectopic female gametophytes within the ovule^33,34^. To determine if the truncation of DCL3 and the unusual distribution of sRNAs could be related with female gametogenesis in *U. gibba* as has been reported for Arabidopsis, we first analyzed female gametogenesis in whole-mounted developing ovules*,* as no descriptions of ovule development have been previously reported for this species. Our results are described and illustrated in Fig. 4c-i and in Additional file 2: Table S4.

As for other species of *Utricularia*^35,36^, the ovule of *U. gibba* is unitegmic (only 1 integument), with a funiculus forming a raphe and merging into a voluminous placenta. The formation of differentiated gametes occurs after the formation of meiotically derived megaspores (megasporogenesis). Subsequent rounds of mitotic divisions give rise to the female gametophyte (megagametogenesis). Megasporogenesis occurs in ovules within ovaries having a diameter of 0.3 to 0.5 mm. Whereas 51.4% (n=142) of pre-meiotic ovules showed a single megaspore mother cell (MMC; Fig. 4c), 42.9% showed from two to six differentiated cells resembling the MMC (Fig. 4d and e). In 5.7% of the ovules examined we could not identify a pre-meiotic precursor. Ovules contained in 0.5-1 mm ovaries had already undergone meiosis and often show a chalazal functional megaspore (FM; Fig. 4f) within which mitotic divisions will give rise to an 8-nucleated syncytium (Fig. 4g) that will cellularize before differentiating into a mature female gametophyte (FG). Although the degeneration of the FG prior to the end of megagametogenesis is not uncommon (13.1%; n=76), in most cases (40.7%; n=76) the ovule contains a FG in which the micropylar region containing the egg apparatus expands outside the integument and grows within the placenta (Fig. 4g and 4h). Interestingly, 22.4% of ovules examined showed supernumerary gametic cells in the chalazal region, independent of the developing FG (Fig. 4h), and containing two independently developing FGs (Fig. 4i), suggesting that supernumerary gametic precursors can give rise to female gametophytes that may or may not originate from a meiotically derived cell. The presence of supernumerary gametic precursor cells and ectopic female gametophytes in *U. gibba* is reminiscent of phenotypes found in Arabidopsis mutants *dicer-like3* (*dcl3*), *argonaute4* (*ago4*), *argonaute9* (*ago9*), *rna-dependent rna polymerase6* (*rdr6*), and *nrpd1a*, all affected in key components of the RdDM pathway.

### *U. gibba* has a reduced levels of DNA methylation

In plants, 24-nt siRNAs from repetitive DNA and TEs that are loaded by AGO4/6 trigger DNA methylation, which results in histone modifications such as the H3K9me2^37,38^. Because of the unusual distribution of 24-nt sRNAs and the low proportion of TEs and other repetitive DNA in *U. gibba*, we decided to explore preliminarily the global DNA methylation patterns in this carnivorous plant using long-read DNA sequencing data with the technology of SMRT-Seq, recently reported^15^. This technology measures each base addition as an interpulse duration (IPD) or retention time ratio. The IPD will depend on whether the new base is incorporated by pairing to a modified or non-modified base in the template and the nature of the modification^39^. Therefore, analysis of the IPDs during SMRT-Seq can allow the identification of 5mC in the DNA template without the need for commonly used bisulfite DNA chemical conversion methods. Since there are no previous reports of using SMRT-Seq data to determine 5mC global methylation in plants, we tested as preliminarily the 5mC identifications in *U. gibba* taking advantage of PacBio data from its genome (http://merlion.scelse.ntu.edu.sg/shares/pbio_HGYDGSKAA23/). Raw SMRT-Seq data was aligned against the reference genome and base kinetics information analyzed using the program SMRT-link V4.0 to identify base modifications and the theoretical IPD value for non-modified bases which is 1 was used.

The genome-wide depth with SMRT sequencing was ~ 70X and the mean coverage for all bases was 34X. We identified 1,590,729 methylated cytosines, which represents 5.3% of the global methylation level. The fraction of total 5mCs context as a proportion of total Cs in that context were 12% for CG, 5% for CHG and 2.3% for CHH (Additional file 2: Fig. S18b), which are lower than those reported for soybean and Arabidopsis^40^. These results suggests that PacBio data could be useful to generate a DNA methylation landscape of plant genomes. Moreover, we decided to explore the distribution of genic DNA methylation levels for all genes (Additional file 2: Fig. S18c).

**Figure 5:**
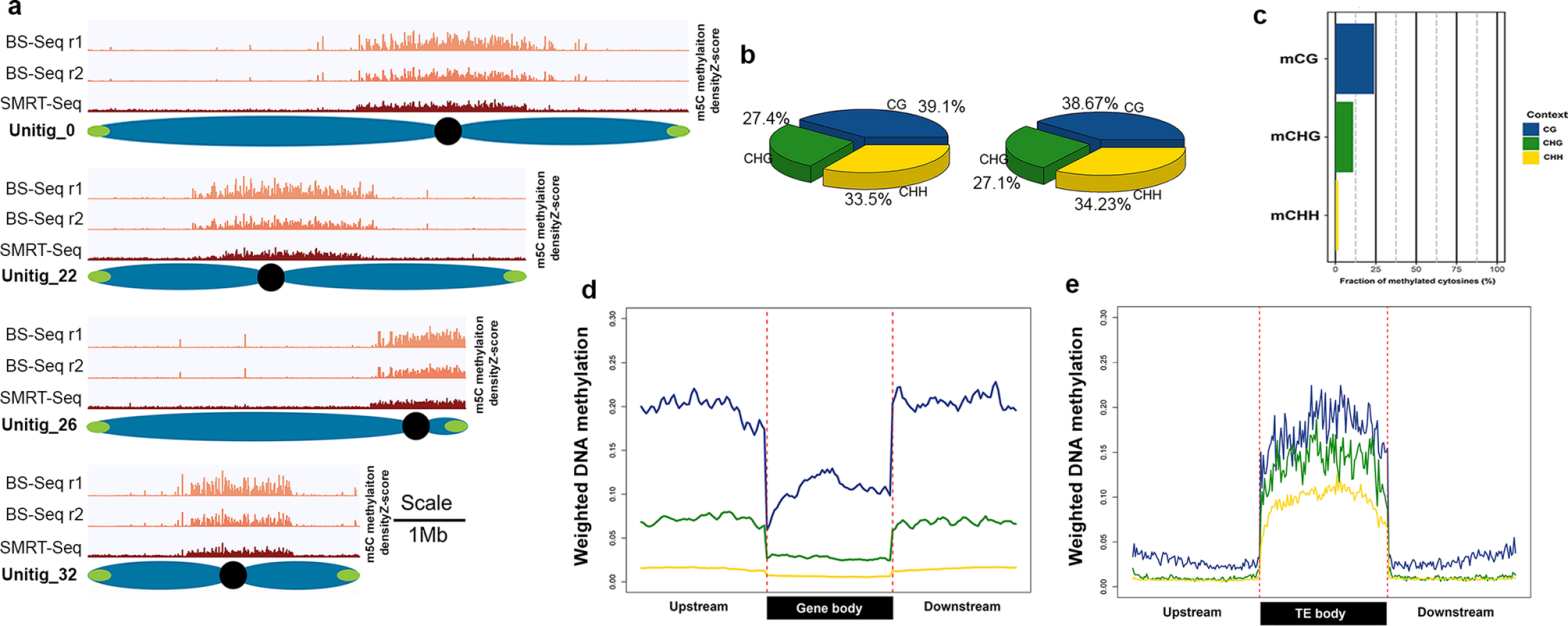
Genome-wide methylation in *Utricularia gibba*. (**a**) Distribution of methylation (5mC) representation only in the complete chromosomes in *U. gibba* for location of pericentromeric regions. Here, the blue light red shows the methylation density in BS-Seq replicates and the red histogram represent the methylation distribution in SMRT-Seq with normalized data in Y-axis representing the 5mC density in windows of 100kb (Scale 1Mb). (**b**) DNA methylation level for each context at the genome level. X-axis shown the methylation level according to Schultz et al., 2012. CG methylation in blue, CHG methylation in green, and CHH methylation in yellow. (**c**) Methylation levels for CG, CHG and CHH context. (**d**) Distribution of DNA methylation around all genes. Methylation representation in all contexts (CG, CHG and CHH) 2-kb upstream and downstream. (**e**) TE DNA methylation representation in all contexts (CG, CHG and CHH) 2-kb upstream and downstream.

To determine whether the level of methylation and the context distribution of 5mC predicted by analysisng SMRT-Seq data, we performed BS-Seq for two replicates of *U. gibba*. The bisulfite conversion rates in both replicates were greater than 99.85% with a mean coverage for base of 26X. After sequencing, clean reads were mapped against the reference genome obtaining around 85% of mapping rate. After base calling, global DNA methylation level was determined to be 6.5%, which is similar to that determined by SMRT-Seq (5.3%). Analysis of the distribution of 5mCs at the chromosome level shows peaks near centromeric regions for both replicates of BS-Seq and for SMRT-Seq (Fig. 5a,b) with very similar distributions. Distribution of 5mC in *U.gibba* was similar to that reported in other plant genomes with one of the lowest global DNA methylation level reported for angiosperms, including that of *Arabidopsis thaliana* that ranges between 7.5 and 9%^11,12^. When comparing the DNA methylation levels for each context (CG, CHG or CHH) related, calculated as weighted methylation levels according to Schultz et al. 2012 (77), the methylation levels were 24% for CG, 10.95% for CHG and 1.8% for CHH (Fig. 5c). The levels of methylation in *U. gibba* were lower than in other plants with larger genomes such is soybean, and interestingly lower than in Arabidopsis, particularly for the methylation level in the CHH context that was about half of that reported fort Arabidopsis^40^. The distribution of genic DNA methylation was evaluated for all genes; low levels of methylation were found in the coding region particularly around translation start/stop sites with the prevalence of CG methylation context and very low CHH methylation levels. DNA methylation genic region of U. gibba (Fig. 5d) is similar to that previously reported for other angiosperms having lower levels of methylation in the coding part particularly near the start and stop codons^12^. Analysis of DNA methylation of TEs showed elevated methylation in all three contexts across the entire body (Fig. 5e), which could explain the TE silencing in this genome.

We performed a chop-qPCR to test DNA methylation patterns obtained by the sequence analysis of PacBio reads. Two different regions in the *Utricularia gibba* Unitig 0 were selected, a gene-poor region (Gp, Pericentromeric) and a gene-rich region; from those two regions, a fragment that contained methylated cytosines overlapping with methylation sensitive enzymes *HaeII* and *HpaII* restriction sites was selected, as a control we growth Utricularia plants in MS media supplemented with 50 µM Zebularine to induce global DNA demethylation (Additional file 2: Fig. S19). Briefly, a decrease of the amplification rate of the selected fragments in the non-Zebularine treated samples compared with the samples treated with Zebularine would mean that the *HaeII* and *HpaII* restriction sites were methylated, and these patterns correlated with those obtained by PacBio sequencing. Our findings suggest that the SMRT-sequencing technology could be used effectively to estimate global methylation patterns in plant genomes and BS-Seq confirm several results obtained with SMRT-Seq.

## Discussion

### Genome rearrangements after WGD in *U. gibba* and their role in ncRNA content

The causes and mechanisms of WGD events and genome fractionation processes that lead to genome expansion and contraction in *U. gibba*, are still poorly understood. Moreover, the consequences of these processes on the repertoire of non-coding RNAs and DNA methylation processes remain obscure. *U. gibba*, a carnivorous plant with an unusually small but dynamic genome that has experienced two relatively recent WGD events, represents a highly illustrative model to study the processes of genome contraction and its consequences on the diversity of ncRNAs, as well as its consequences on DNA methylation. Our analysis of ncRNAs provides insights into the distribution and repertoire of ncRNAs in carnivorous plants. One of our interesting observations is that in contrast to other angiosperms, such as Arabidopsis, maize, and tomato, for which lncRNA *loci* are preferentially located in centromeric regions, in *U. gibba* lncRNA *loci* are well distributed across the genome but with a lower density in centromeric regions (Fig. 2). Centromeres are genomic sites of spindle attachment essential for ensuring proper chromosome segregation during cell division. Despite their recognized functional importance, centromeres are not well defined at the sequence level in eukaryotic genomes except for some small fungal genomes^41^. In general, centromeres are accepted to be composed of high-copy tandem satellite repeats and/or the presence of centromeric chromoviruses, a lineage of Ty3/gypsy retrotransposons. Also, these sequences may be merely parasitic and tend to accumulate in recombination-poor centromeric regions to escape negative selection^42^. The low density of lncRNAs observed in the putative centromeric regions of the *U. gibba* genome could be related to the absence of both satellite repeats and paucity of centromeric chromoviruses in these regions^15^. Centromeres without long arrays of satellite DNA have been referred to as evolutionarily new centromeres^43^. It is possible that after the last WGD event in *U. gibba*, genome fractionation resulted in the formation of neo-centromeres targeted by few lncRNAs. These findings are consistent with the notion that histone modification, in the form of stable, self-propagating chromatin states, rather than sequence specific structural features, define a functional centromere.

### A truncated *DCL3* gene correlates with the distribution of sRNAs and impact the developmental biology of *U. gibba*

TE proliferation shapes genome sizes among eukaryotes. In plants, one of the mechanisms to control this expansion is through TE silencing via 24-nt siRNAs^44,45^. In the siRNA biosynthesis pathways, at least three RNA-dependent RNA polymerases (RDR1, RDR2, and RDR6) are functional in plants^29,30^. RDR2 works with DCL3 to form chromatin associated siRNAs (mainly 24-nt) that are involved in sRNA mediated DNA methylation^8^. As expected from its known role in the biogenesis of 24-nt small RNAs, Arabidopsis and maize mutants are defective in RDR2 or DCL3 show a severe reduction in the production of the 24-nt size siRNA class, whereas the 21-nt class (miRNA) is not affected and indeed becomes overrepresented^46,47^. In rice, DCL3 mutations also affect the production of 24-nt siRNAs associated with TEs^48^. Here, we report that *U. gibba* has an unusually a sRNA size distribution more similar to early-branching land plants and gymnosperms than to other angiosperms and low content of 24-nt sRNAs. (Fig. 3a). However, the accumulation of 24-nt siRNAs in *U. gibba* is similar to *dcl3* mutants in Arabidopsis, rice and tomato^46,48,49^, which correlates well with the presence in *U. gibba* of a truncated form of DCL3 (Fig. 4a,b). The loss of a fully functional DCL3 in *U. gibba* may be responsible for the reduction in the 24-nt class of sRNAs. It is possible that the loss of a fully functional DCL3 in *U. gibba* might have originally reflected lower selective pressure to silence fewer TEs. However, it may happen that these low levels of DNA methylation also are coupled to active demethylation events mainly by the family of 5mC DNA glycosylases such are REPRESSOR OF SILENCING 1 (ROS1), TRANCRIPTIONAL ACTIVATOR DEMETER (DME), DEMETER-LIKE PROTEIN 2 (DML2) and DEMETER-LIKE 3 (DML3)^50^, all of which we found transcriptionally active in *U. gibba*. Furthermore, we report the first quantitative analysis of female gametogenesis in a member of the *Utricularia* genus and these developmental ovule defects have not been reported previously for *Utricularia* species^36,51^ and this study reveals severe abnormalities in the ovule development of *U. gibba*.

Animal Dicer and plant DCL proteins dimerize at their RNase III domains^52,53^. It is possible that the truncated DCL3 acts as an interfering subunit in the DCL protein complex to generate a dominant negative phenotype^54^ or simply that has no function. As hypothesis for the first, this dominant negative effect could suppress the production of the 24-nt class of siRNAs accompanied by defects that include the absence of a female gametophyte that leads to partial female sterility, or the differentiation of female ectopic precursors that give rise to supernumerary gametophytes. It would be interesting to further study the biological implications of the DCL truncation in *U. gibba*, as it could represent an initial step in the evolution of RdDM pathway and further developmental and genetic studies are required to determine if the latter could eventually result in variable frequencies of unreduced gamete formation or polyploidization events.

### SMRT-Seq as an alternative to decipher plant methylomes

All plant methylomes sequenced to date have been generated by BS-Seq^39^. In recent years, however, new techniques for sequencing has generated the possibility of direct DNA base modification detection without any chemical conversion^39^. Recently, genome-wide DNA methylation on N-6 adenine using SMRT-sequencing technology was reported for some animals and plants^55–57^. Our results of *U. gibba* BS-Seq support the use of SMRT sequencing technology to determine 5mCs on whole genomes. At least in qualitative terms, SMRT technology produces whole-genome 5mC data similar in the percentages of methylated cytosines, the proportions of methylation context (CG, CHG and CHH), genome-wide 5mC distribution to those obtained by bisulfite sequencing. Similar findings we obtain with SMRT-Seq preliminarily data and BS-Seq in the model plant *A. thaliana,* which strongly support our results (Additional file 2: Fig. S20). Some of the methylation variability between identification methods can be explained by de dynamic DNA methylation among cells, individuals or natural variation^58–60^, bias in libraries preparation strategies^61^ or the differences between methods. Both BS-Seq and SMRT-Seq are based on the capture of 5mC from a pool of diploid cells making difficult to estimate the influence of 5mC modifications on gene expression. Here, the approach of single-cell sequencing would be the suitable method for understand this relationship. BS-Seq has so far been the standard for methylome determination and has been quite useful to obtain the DNA methylation landscapes, however, SMRT-Seq could be an alternative to directly generate DNA methylation patterns without additional chemical DNA treatment. Further detailed analyses comparing the two methods in specific genomic regions will be required to ascertain the extent to which SMRT technology can generate robust quantitative data on 5mC DNA modifications, the fact that methylation patterns can be established linearly for long-reads could serve to establish this method to decipher methylomes.

### Lower DNA methylation levels in *U. gibba* in comparison with other plant genomes

Focusing on canonical genes in RdDM pathway, *de novo* DNA methylation involves Pol-IV, Pol-V and several key genes^8,25^. However, we could not uncover a direct AGO6 ortholog, and the DCL3 ortholog is truncated in its catalytic domains. In Arabidopsis *dcl3* and *ago6* mutants have reduced DNA methylation levels at RdDM targets^62,63^. Moreover, AGO4 and AGO6 in Arabidopsis are not as redundant as was originally thought. Indeed, physical interactions demonstrate that they have specific functions: AGO4 is colocalized with Pol-II, and AGO6 interacts with Pol-V^63^. Global DNA methylation of *U. gibba* is around 6.5%, among the lowest proportions reported for plant species, which range between 5% in *Theobroma cacao* to 43% in *Beta vulgaris*^11^. Our results suggest that the reduced level of 24-nt siRNAs in *U. gibba* could be due to the presence of a truncated DCL3 and/or the absence of AGO6, in turn impacting DNA methylation, mainly in methylation level of CHH context which in this work reported as reduced compared with *A. thaliana* and other angiosperms.

## Conclusions

First, this study on *Utricularia gibba*, a plant with a remarkably small genome size that has undergone drastic genome contraction after two recent events of WGD, sheds light on noncoding RNA landscape and its impact on DNA methylation in plants with compact genome size, which provides valuable information for future studies of plant genome engineering and functional genomics. Second, they provide a genome-wide picture of the DNA methylation in *U. gibba*, estimated by long-read sequencing with SMRT technology of PacBio and tested in the model plant *A. thaliana*, which also confirm that PacBio data potentially provide an overall picture of methylation status. Third, they report correlation among truncated DCL3-unusual repertoire of sRNAs-low global DNA methylation which may suggest an impact in the reproductive developmental biology of this plant and altered canonical RdDM pathway. Finally, we propose that alterations in the RdDM pathway could be the result a relaxed DNA methylation control of the low content of transposable elements in *U. gibba*.

## Methods

### Plant material

*U. gibba* was collected in 2006 Umécuaro village in the municipality of Morelia, México. Specimens were taxonomically determined by Dr. Enrique Ibarra according to the key in Mickel & Smith (2004) and voucher specimens were deposited at MEXU herbarium, UNAM. Since then the plant material has been propagated in an aquarium and samples can be ontained from eother the MEXu herbarium or from Dr. Luis Herrera-Estrella at Cinvestav in México. The plant tissue used in this report is derived from the same specimen used for sequencing the *U. gibba* genome (13).

Collected plants were grown in sterile tissue culture with liquid media MS (0.25X) at 22 C with 16 hours light and 8 hours of darkness. Subcultures, at day 14 of growth, were used in our experiments. We employed the following growth conditions and treatments: nutrient deficiency, exposure to plant hormones, high and low temperatures, continuous illumination and darkness, treatment with acidic and alkaline pH, osmotic stress, and saline stress. Traps and plant tissue were collected separately after short (48 and 72 hours) and long (7 and 14 days) time treatments for total RNA extraction and subjected to RNA-Seq (Additional file 1: Dataset S1).

### Total RNA extraction and sequencing

Total RNA from three independent biological replicates for all the analyzed treatments was isolated using the TRIzol reagent (Life technologies), except for trap tissue, which was isolated using the Direct-zol RNA kit (Zymo Research) because this protocol optimizes RNA extraction for low tissue quantities and improved RNA quality for these samples. Twelve RNA-Seq libraries were prepared with the TrueSeq (Illumina technologies) kit and sequenced by non-directional single-end mode.

### Mapping reads and lncRNAs identification

Trimmed reads were mapped against the latest available version of the *U. gibba* genome (https://genomevolution.org/coge/GenomeInfo.pl?gid=28800). To potentiate the identification of lncRNAs in *U. gibba* we decided to merge mapped reads for each library in a single file to obtain a core transcriptome assembly with Trinity v2.0^64^. The identification works with three major filters: I) One of the main features for lncRNAs is size, then during the assembly the transcripts with less than 200 nucleotides in size were cut-off. The first step was to search protein orthologs against a database of non-redundant eukaryotic proteins^14^, this step eliminates those transcripts that encode for proteins. II) Putative lncRNAs transcripts were filtered finding homologs with housekeeping RNAs such are transfer-RNAs (tRNAs), ribosomal-RNAs (rRNAs), micro-RNAs (miRNAs) and other types of structural RNA using the database Rfam v12.0^65^ and sequence homologs with precursors of small RNAs from *U. gibba* dataset. III) Third step relays in the evaluation of the coding potential of the remaining transcripts using the tools CPC2^66^ and CPAT v1.2.4^67^ using the corrected training for *U. gibba* model. The lncRNAs identification is described and summarized in Additional file 2: Fig. S1.

### Small RNA sequencing and annotation

RNA was extracted independently from each sample and then equimolarly pooled to produce three small RNA libraries (Additional file 1: Dataset S1). Small RNA libraries were constructed using the TrueSeq Small RNA kit (Illumina Technologies) and sequenced using NextSeq (Illumina Technologies) using a 50-nucleotide single end runs. The reads were filtered and processed with the kraken toolset^68^. The sequences after adapter trimming and quality filters were selected to further analysis. The reads were mapped against the *U. gibba* reference genome and the alignments were filtered and then run in ShortStack V2.0^23^ The readings were filtered and processed with the Kraken toolset (Davis et al., 2013). ShortStack-count mode was used to find relative small RNA abundances of *de novo*-identified sRNAs *loci*. The sRNAs reads were mapped against mature sequences in the miRNA database miRBase V22.0 (blastn –word_size 7 –dust no –identity 90 –evalue 1000).

### Phylogenetic analysis

Were identified the homologs among plant genomes in Phytozome version 12.1 (http://phytozome.jgi.doe.gov/;^69^). For all sequences, homology analysis was performed (Blastx E-value < 0.001, Bit-Score > 70). Additionally, we performed a bidirectional blast analysis against Arabidopsis protein database from TAIR V10 (www.arabidopsis.org) using previous parameters. Protein multiple alignments were made using MAFFT V7.0^70^ and trimmed by trimAL^71^. Finally, the best model fit was selected to execute the phylogenetic inference with ProtTest V3.0^72^. The Bayesian phylogenetic reconstruction was executed using the software MrBayes V3.2^73^ with 300000 generations. Also, likelihood phylogenetic reconstruction was estimated using RAxML back box on website CIPRES (https://www.phylo.org/) with GTR + G model and 1000 cycles for bootstrapping.

### Determination of selection pressure in the DCL3 clade

The Fixed Effects Likelihood (FEL) analysis^74^ allowed us to identify signatures of natural selection strength in the DCL3 sequences from *U. gibba* and its comparison against *A. thaliana* and *M. guttatus* (Additional file 1: Dataset S1). The analysis was performed independently for each sequence taken as background all sequences contained in the clade DCL3. We follow the procedure reported before in the ANT transcription factors analysis along plant phylogeny^75^.

### Cytological analysis of ovule development

For cytological examination of ovules, whole flowers were harvested and fixed in formalin acetic acid-alcohol solution (40% formaldehyde, glacial acetic acid, 50% ethanol; in a 5:5:90 volume ratio) for 24 hours at room temperature, and subsequently stored in 70% ethanol at 4° C. Fixed ovaries were dissected with hypodermic needles (1 mm insulin syringes), cleared, and observed by differential interference contrast microscopy using a Leica DMR microscope.

### Identification of 5mC with SMRT-sequencing and validation

Raw data was obtained from two sources: 1) The PacBio *U. gibba* SMRT sequencing data were provided by the authors of genome sequencing paper (http://merlion.scelse.ntu.edu.sg/shares/pbio_HGYDGSKAA23/) (4). 2) The Arabidopsis PacBio data was downloaded from PacBio public database for ler-0 ecotype (http://datasets.pacb.com.s3.amazonaws.com/2014/Arabidopsis/list.html). Each PacBio SMRT-cell provides raw data in bax.h5 format, these raw data were aligned against plant reference genome (*A. thaliana* or *U. gibba*) using pbaling program in base modification mode from SMRT-Link program designed by PacBio. For each case, to obtain more coverage and depth were merged the SMRT-cells (10 SMRT-cells for *U. gibba* and 40 SMRT-cell for Arabidopsis) and with loadPulses scripts (SMRT-link) the polymerase kinetics information for each base was obtained as average of IPD-ratio. Finally, the modification of cytosine was identified using ipdSummary.py script (SMRT-link).

The theoretical IPD value for non-modified bases is 1. We found that for non-modified cytosines the median of IPD ratio was 1.02 (S=0.19), whereas for modified cytosine (5mC) the median was 1.6699 and the mean 1.84, whereas for high confidence modified cytosine (5mC) the median was 1.89 (S=0.33) and average IPD was 2.02, which corresponds to the expected IPD for 5mC. Raw data in bax.h5 format was aligned using pbaling in base modification mode. Polymerase kinetics information was further loaded after alignment using loadPulses scripts. Finally, 5mCs were identified using the ipdSummary.py script. We used a filter of 15X coverage to select for 5mCs. Parts of the analysis were done using customized scripts in R, MySQL, and Perl. To validate our results, two genomic regions were selected from an entire chromosome, Unitig_0. Vegetative tissue explants were grown for 14 days and were transferred to new media with or without 50 µM of Zebularine for 8 days. DNA from this experiment was collected. The DNA was either digested or not with the methylation sensitive restriction enzymes *HaeII*. A quantification was performed using Chop-qPCR (Additional file 2: Fig. S20).

### Whole-genome bisulfite sequencing

Genomic DNA from *U. gibba* fresh tissue 14-days old was grinded with liquid nitrogen and purified with DNeasy Plant Maxi Kit (Qiagen). For the library preparation, DNA samples were fragmented into 200-400 bp using a sonicator S220 (Covaris). Then DNA fragments were blunt ended and, a dA 3′-end addition was performed prior to sequencing adapter ligation. Illumina methylated adapters were used according to the manufacturer’s instructions (Illumina). The DNA fragments were bisulfite treated with EZ DNA methylation Gold Kit (Zymo Research). The final DNA library was obtained by size selection and PCR amplification. High-throughput pair-end sequencing was carried out using the Illumina HiSeq 2500 system according to manufacturer instructions. The Clean reads were aligned to reference genome using Bismark software^76^. To identify the true methylated sites, methylated and unmethylated counts at each site from Bismark output was tested by binomial distribution. Genome-wide weighted DNA methylation was calculated from all aligned data by dividing the total number of aligned methylated reads (coverage>=3) to the genome by the total number of methylated plus unmethylated reads as detailed in Schultz *et al*., 2012^77^.

### Comparison between sequencing platforms in Arabidopsis and *U. gibba* methylomes

Once obtaining the 5mC identification with SMRT-Seq in Arabidopsis, we used previous bisulfite sequencing dataset from our group^78^ to compare both methods. We calculated the cytosine coverage per chromosome (the number of cytosines identified by sequencing method) with bisulfite sequencing and SMRT-Sequencing to compare the depth for each identification method. Also, the percentages of 5mC identification per chromosome (number of cytosines methylated/total of cytosines) were calculated. The methylation contexts were calculated from motif (3-bases) generated. Finally, the comparisons were reported as metaplot graphs of 5mC density per chromosome corrected by Z-score analysis and barplot for each library of Bisulfite sequencing and SMRT-sequencing.

### Correlation analysis

The datasets provided by other groups for the normalization of the SMRT-Seq 5mC identification were for SMRT-Seq of the Arabidopsis ecotype *Landsberg erecta* (*Ler-0*) and for BS-Seq of Arabidopsis *Columbia* (*Col*-0). To correlation analysis, we used syntenic regions between *Col*-0 and *Ler*-0 provided from previous reports, where one of these analyses was obtained with best assembled genomes^79,80^. The syntenic regions were obtained with last assembled genomes applying SyRI, a program that reports syntenic regions of genomes^81^. Only large syntenic regions detected were used to assign them in the corresponded density window and different IPD cut values were used for the 5 chromosomes of *Arabidopsis thaliana* genome. These syntenic regions were used to study the tracks of density for both, BS-Seq and SMRT-Seq. In summary, data consist of methylation density estimation by BS-Seq and SMRT-Seq in 604 syntenic genomic windows, each one covering 100,000 bp for a total genome coverage of 60.4 Mb. Pearson correlation coefficient, r, was calculated for the whole set of syntenic regions to estimate correlation at genomic level as well as dividing the syntenic set by chromosome of origin. The normalized methylation density data were plotted in R. In each case the null hypothesis of zero correlation was tested.

### Ethics approval and consent to participate

Sampling site of *Utricularia gibba* is not classified as protected natural area and U. gibba is not included in the Mexican Official Norms (NOM-059-SEMARNAT-2010) as endangered species. Specifically, *U. gibba* was collected with prior consent of landowner (Mr. Gerardo Negrete Negrete). All experiments were carried out according to national and international guidelines for research in plant biology.

## Supplementary Information

Supplementary Information 1.

Supplementary Information 2.

Supplementary Information 3.

Supplementary Information 4.

## Data Availability

All files containing reads and quality scores were deposited in the National Center for Biotechnology Information (NCBI) archive [BioProject: PRJNA526734; SRA: SRR8717102, SRR8717105, SRR8717107, SRR8717103, SRR8717106, SRR8717097, SRR8717101, SRR8717104, SRR8717093, SRR8717098, SRR8717099, SRR8717100 and BioProject: PRJNA633566; SRA: SRR8717102, SRR8717105].
