# Supplementary material for: Atypical DNA methylation, sRNA-size distribution, and female gametogenesis in Utricularia gibba
Source: Sci Rep. 2021 Aug 3;11:15725. doi: 10.1038/s41598-021-95054-y (PMC8333044; doi:10.1038/s41598-021-95054-y)
Supplement: Supplementary file 2 — Supplementary Information 2. [file 41598_2021_95054_MOESM2_ESM.docx]

**Atypical DNA methylation, sRNA-size distribution, and female gametogenesis in the compact genome of *Utricularia gibba****.*

Cervantes-Pérez, Sergio Alan; Yong-Villalobos, Lenin; Florez-Zapata, Nathalia M. V.; Oropeza-Aburto, Araceli; Rico-Reséndiz, Félix; Amasende-Morales, Itzel; Lan, Tianying; Martínez, Octavio; Vielle-Calzada, Jean Philippe; Albert, Victor A. and Herrera-Estrella, Luis.

**Additional file 2**

The following Additional file 2 is available for this article:

**Fig. S1** Workflow of lncRNAs identification.

**Fig. S2** Intergenic lncRNAs identification in some plants.

**Fig. S3** Small RNAs size distribution in the three libraries of sRNA-Seq.

**Fig. S4** Known and novel miRNAs loci in comparison with miRBase.

**Fig. S5** Proportion of sRNA reads found for each miRNA family in the three sequenced libraries.

**Fig. S6** Annotation of sRNAs loci.

**Fig. S7** Distribution of Polymerase (IV and V) holoenzymes in plants.

**Fig. S8** NRPD4 phylogenetic tree.

**Fig. S9** AGO5 clade homolog identification.

**Fig. S10** AGO2/3/7 clade in the *U. gibba* phylome.

**Fig. S11** AGO6/4/8/9 clade in the *U. gibba* phylome.

**Fig. S12** AGO1/10 clade in *U. gibba* phylome.

**Fig. S13** Phylogenetic tree of AGO family in *Viridiplantae*.

**Fig. S14** Phylogenetic analysis of DCL and RDR proteins.

**Fig. S15** Conserved protein domains among DCL3 for some angiosperms.

**Fig. S16** Multiple Sequence Alignment of some DCL3 proteins.

**Fig. S17** Ug*DCL3* in the transcriptomic datasets and RACE-PCR alignment.

**Fig. S18** Methylation identification with SMRT-Seq.

**Fig. S19.** Experimental validation of Pac-Bio methylome data.

**Fig. S20** Comparison between m5C identification in *A. thaliana* with BS-Seq and SMRT-Seq.

**Table S1** lncRNAs features. Summary of lncRNAs size and exon number.

**Table S2** List of microRNAs identified by Shortstack.

**Table S3** Canonical genes involved in miRNAs biogenesis.

**Table S4** Ectopic gametic precursors and female gametophytes in the ovule of *Utricularia gibba*.


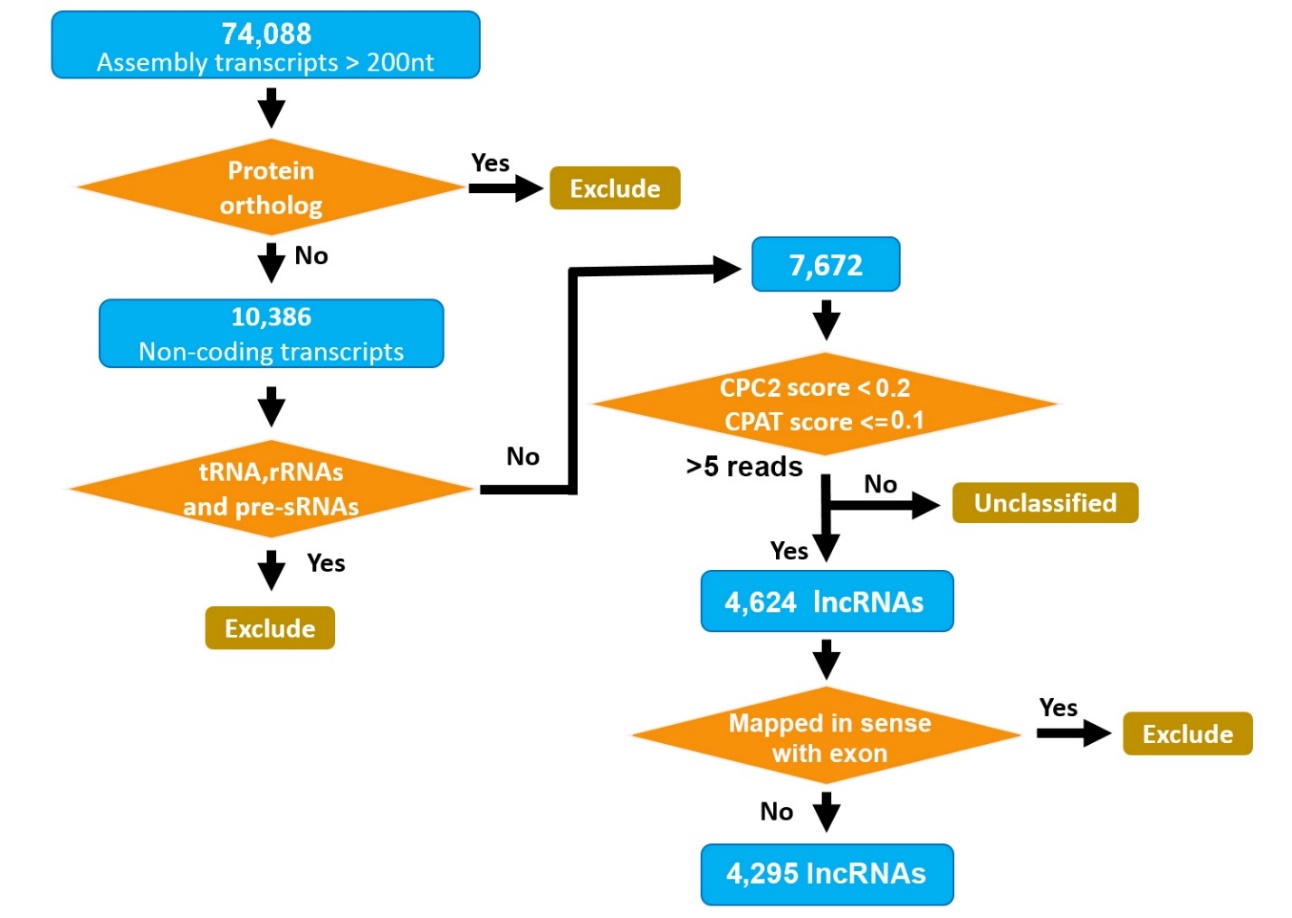


**Fig. S1. Workflow of lncRNAs identification**. Schematization for the identification of lncRNAs in this work. In the workflow, the input/output are representing by blue oval shapes, the arrows are the connector lines that show relationships between the representative shapes, the orange diamonds (which indicates a decision) are the key filters, and brown ovals that are connected to a decision represents all the exclude. A summary with the key steps are described following: the first input of assembled transcripts with size ≥ 200-nt; a decision for protein orthologs must be taken by sequence homology against plant protein database where exclude those transcripts potentially proteins, then the output/input are the transcripts that not found match with proteins, and a second decision implicates the elimination for the similar transcripts to housekeeping and structural transcripts and maintains these without similarity which is the input for the third decision. The final decision calculates the coding potential for a protein in a form of functional ORF eliminating the potentially coding proteins and retaining the final number of putative lncRNAs in *U. gibba* transcriptional landscape.


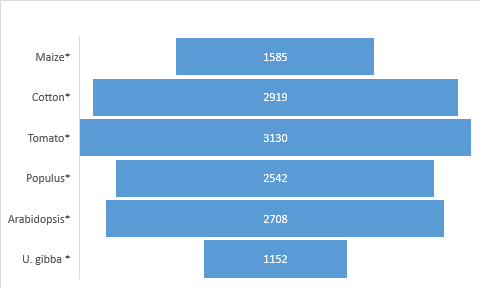


**Fig. S2. Intergenic lncRNAs identification in some plants**. A comparison of intergenic lncRNAs identification for plants only at genomic level searched in the reports for each one of them. For some cases only high confidence lncRNAs were used. The data were obtained from the follow reports: Maize (4), Cotton (5), tomato (6), Populus (7), Arabidopsis (8) and U. gibba (this work).

**
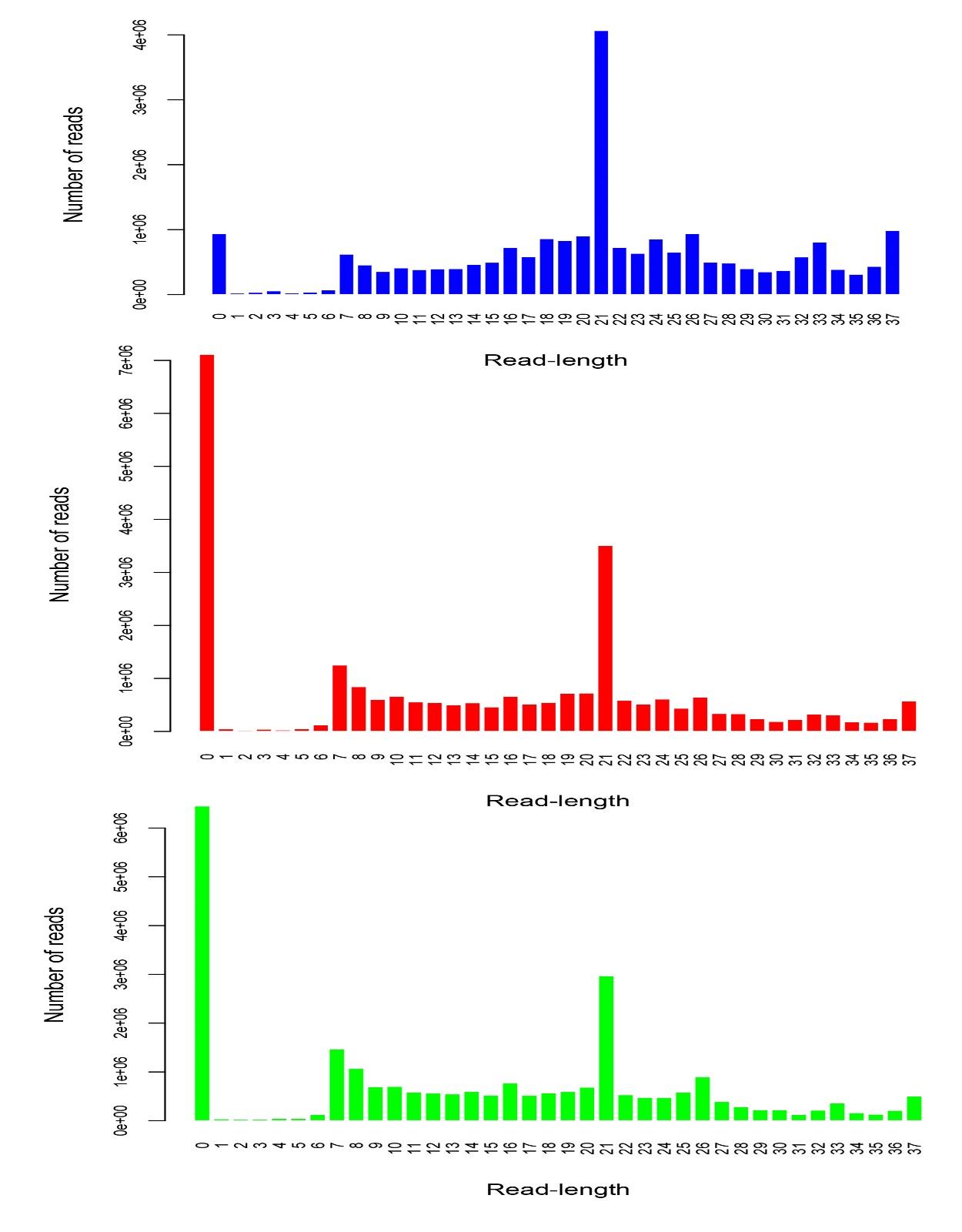
**

**Fig. S3. Small RNAs size distribution in the three libraries of sRNA-Seq.** The distribution of reads size in the small RNA-Seq libraries in *U. gibba* are show in different barplots. Blue barplot represents the nutrients library, the red barplot shows the plant hormones mix library and green barplot represents other conditions library. In all barplot, the X axis is for size in the range of 0 to 37-nt and Y axis show the number of reads per each size.


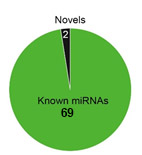


**Fig. S4. Known and novel miRNAs loci in comparison with miRBase.** From 80 miRNA loci predicted by ShortStack (17), 69 miRNAs loci were identified with sequence homologs in miRBase database for other organism and only for 2 miRNAs loci evidence not found in other plant.


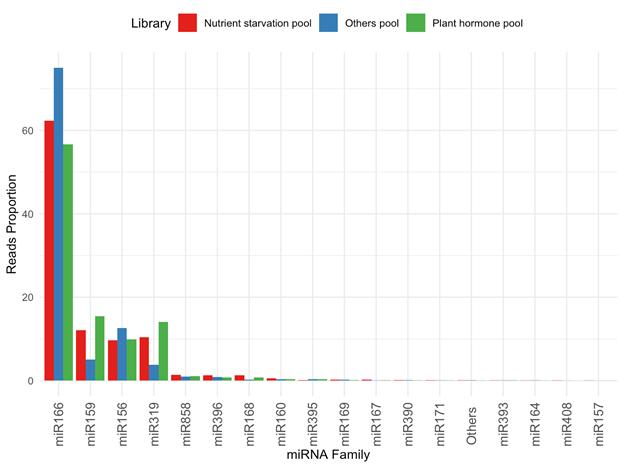


**Fig. S5. Proportion of sRNA reads found for each miRNA family in the three sequenced libraries.** The 17 miRNA families found in *U. gibba* are show in the X axis ordered by descendent proportion of reads (Y axis) the different colors represents the abundance of each miRNA family per library sequenced, nutrients library (red barplot), plant hormone treatments (green barplot) and other conditions library (blue barplot).


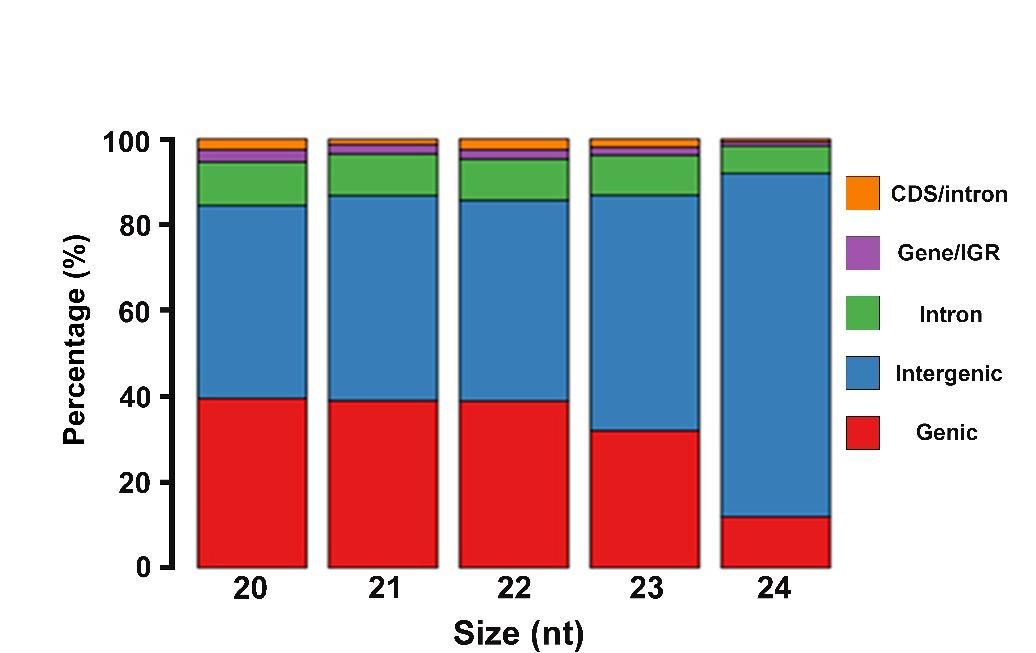


**Fig. S6. Annotation of sRNAs loci.** The distribution of percentage of mapped reads in a range between 20-nt to 24-nt in size for sRNA loci compared with their respective genome annotation. X axis represents the sRNA sizes in nucleotides, the different colors show the genome annotation, and the stacked plot shows the percentage corresponding for each group.


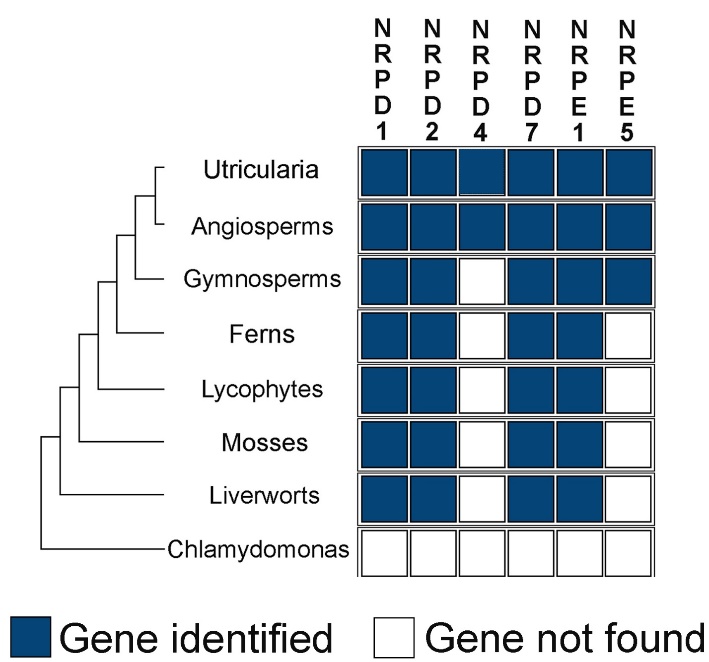


**Fig. S7. Distribution of Polymerase (IV and V) holoenzymes in plants.** An illustrative cladogram with representative plants in each clade is shown and taken from a recent report (19). The modified figure including *U. gibba* (an angiosperm) show the presence (blue square)/absence (white square) of the different subunits of polymerases in plants.


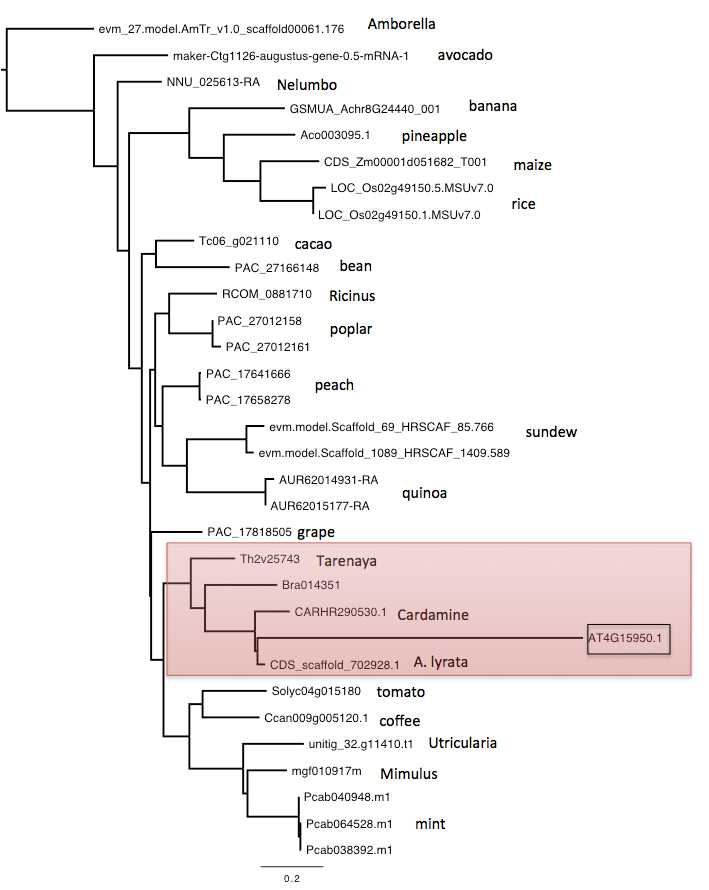


**Fig. S8. NRPD4 phylogenetic tree.** Maximum likelihood tree for NRDP4 homolog genes in some angiosperms (Amborella, avocado, nelumbo, banana, pineapple, maize, rice, cacao, bean ricinus, poplar, peach, sundew, quinoa, grape, Tarenaya, Cardamine, *A. thaliana*, *A. lyrata*, tomato, coffee, Utricularia, Mimulus, and mint). The red rectangle shows NRPD4 for *A. thaliana* and other members of Brasicaceae where *A. thaliana* is divergent even *A. lyrata*. Numbers beside nodes are bootstrap support values showing confidence.

**
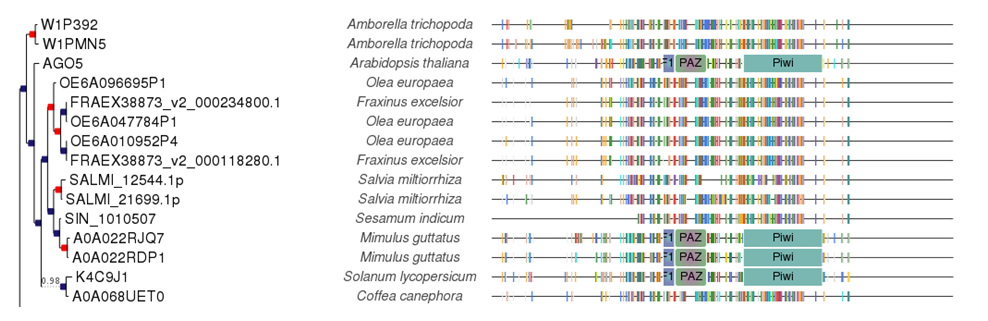
**

**Fig. S9. AGO5 clade homolog identification.** Phylogenetic tree representing the *U. gibba* phylome in the Phylome DB to find the homolog of AGO5 protein.


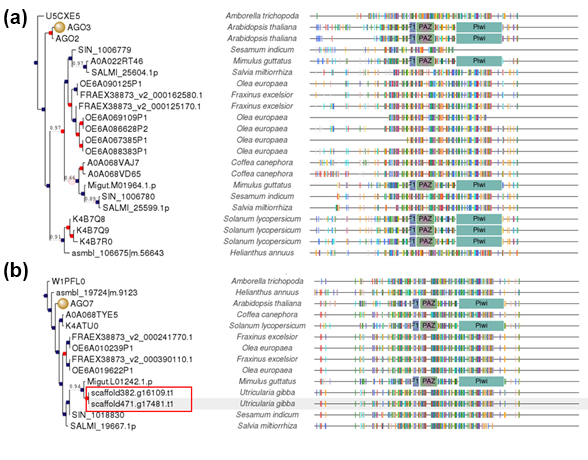


**Fig. S10. AGO2/3/7 clade in the *U. gibba* phylome. (a)** Phylogenetic tree representing the evolutionary history of AGO2, AGO3. **(b)** Phylogenetic tree representing the evolutionary history of AGO7.


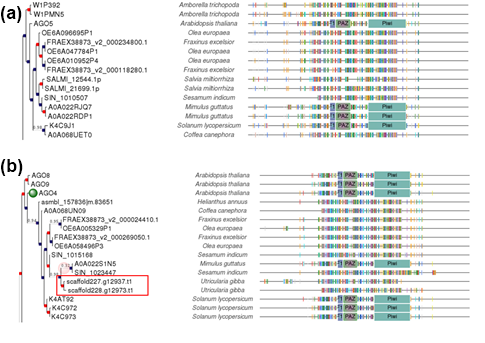


**Fig. S11. AGO6/4/8/9 clade in the *U. gibba* phylome**. **A** Phylogenetic tree representing the evolutionary history in AGO4, AGO8, AGO9 in Phylome DB and **B** evolutionary history of AGO4.


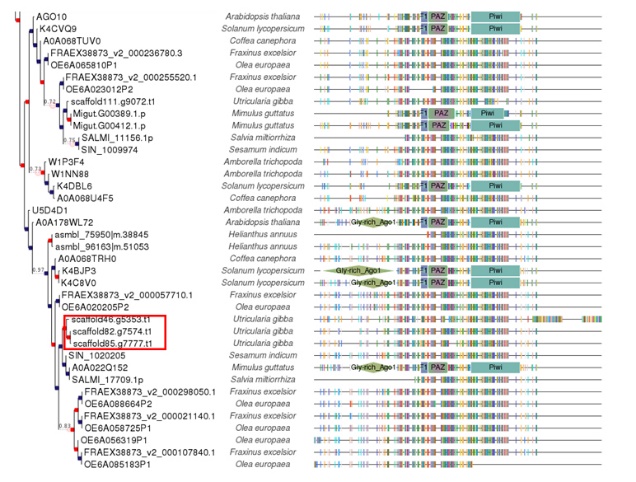


**Fig. S12. AGO1/10 clade in *U. gibba* phylome**. Phylogenetic tree representing the evolutionary history of AGO1 and AGO10 in Phylome DB.


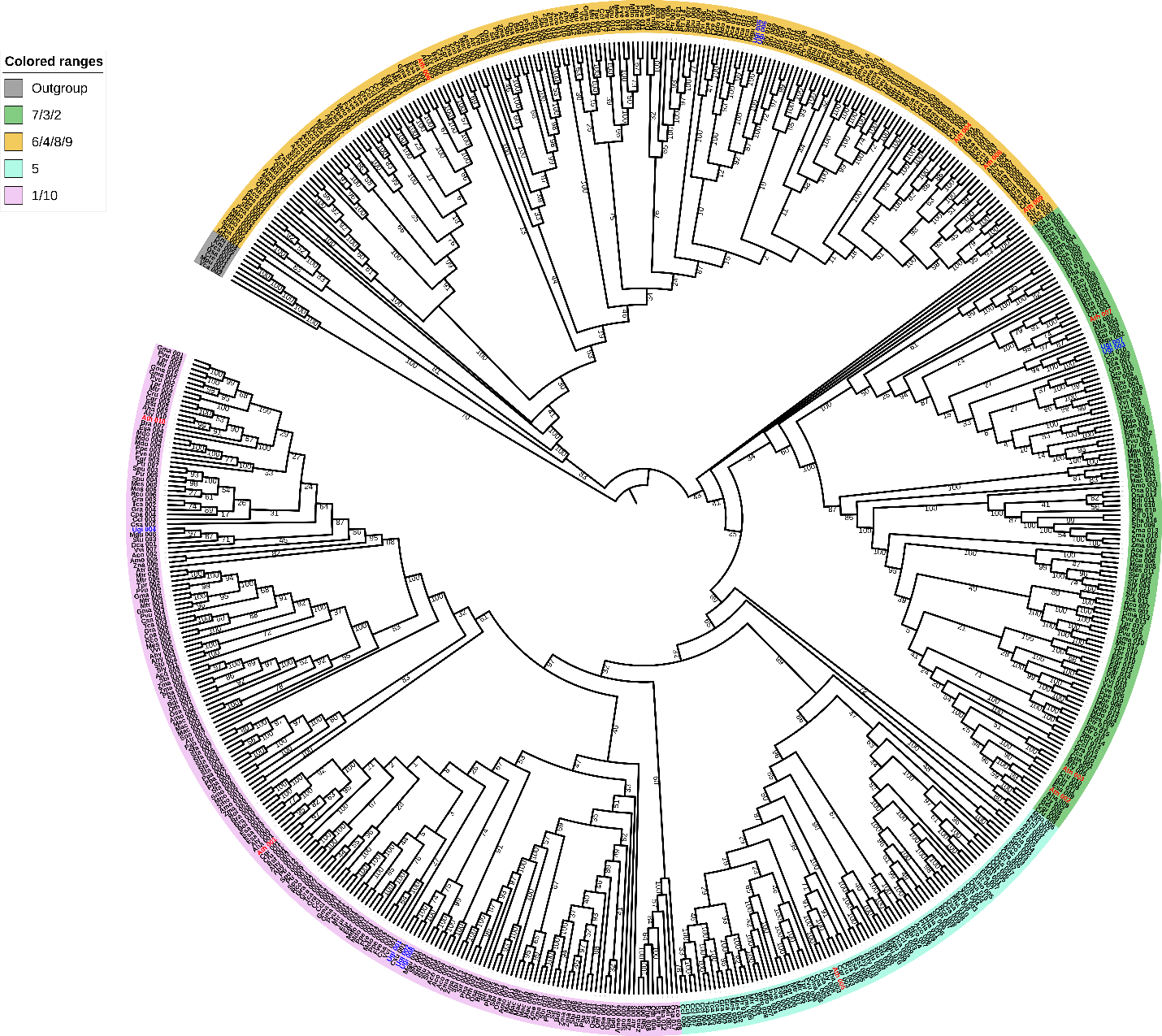


**Fig. S13. Phylogenetic tree of AGO family in Viridiplantae**. Phylogenetic reconstruction of AGO proteins for Viridiplantae to identify AGO in *U. gibba*. A maximum likelihood tree (ML + GTR + bootstrapp=1000) for *AGO1, AGO2, AGO3, AGO4, AGO5, AGO6, AGO7, AGO8, AGO9, AGO10* and *AGO-like* genes from representative genomes of land plants. The sequences from *Charophyte green algae (Coccomixa subellipsoidea, Volvox carteri, Dunaliella salina and Chlamydomonas reinhardtii)* are clustered as out group including only AGO-like genes. Moreover, all AGO sequences from land plants form four clades which are named after with th*e AGO* genes from *A. thaliana (*yellow clade *AGO 6/4/8/9,* green clade *AGO 7/3/2,* cyan clade AGO5 *and* purple *AGO1/10,* respectively*).* Blue flags represent the AGO identified in Arabidopsis and the red flags show the specific AGO for *U. gibba*. Numbers beside nodes are bootstrap support values showing confidence.


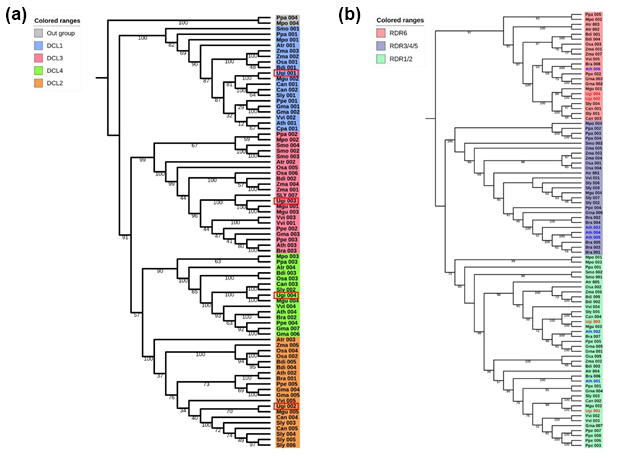


**Fig. S14. Phylogenetic analysis of DCL and RDR proteins. A** Maximum likelihood (ML + GTR + bootstrap = 1000) tree for RDR1, RDR2, RDR3, RDR4, RDR5 and RDR6 from representative genomes of land plants. The Phylogenetic reconstruction reveals an arrangement of three clades which are named after with the *RDR* genes from *A. thaliana (*light red clade *RDR6,* light blue *RDR 3/4/5* and light green *RDR 1/2,* respectively*).* Blue flag indicates the *RDR* sequences which belong to *Arabidopsis thaliana* whereas the flags in red point to *Utricularia gibba* sequences*.* Numbers beside nodes are bootstrap support values showing confidence*.* **B** A maximum likelihood (ML + GTR + boostrap = 1000) tree fo*r DCL1, DCL2, DCL3 and DCL4* from representative genomes of land plants*.* The Phylogenetic reconstruction reveals an arrangement of three clades which are named after with the DCL proteins from *A. thaliana* (Light red clade DCL4, light purple clade DCL2, green clade DCL3 and Blue clade DCL4, respectively). Blue flag indicates the *RDR* sequences which belong to *Arabidopsis thaliana* whereas the flags in red point to *Utricularia gibba* sequences*.* Numbers beside nodes are bootstrap support values showing confidence


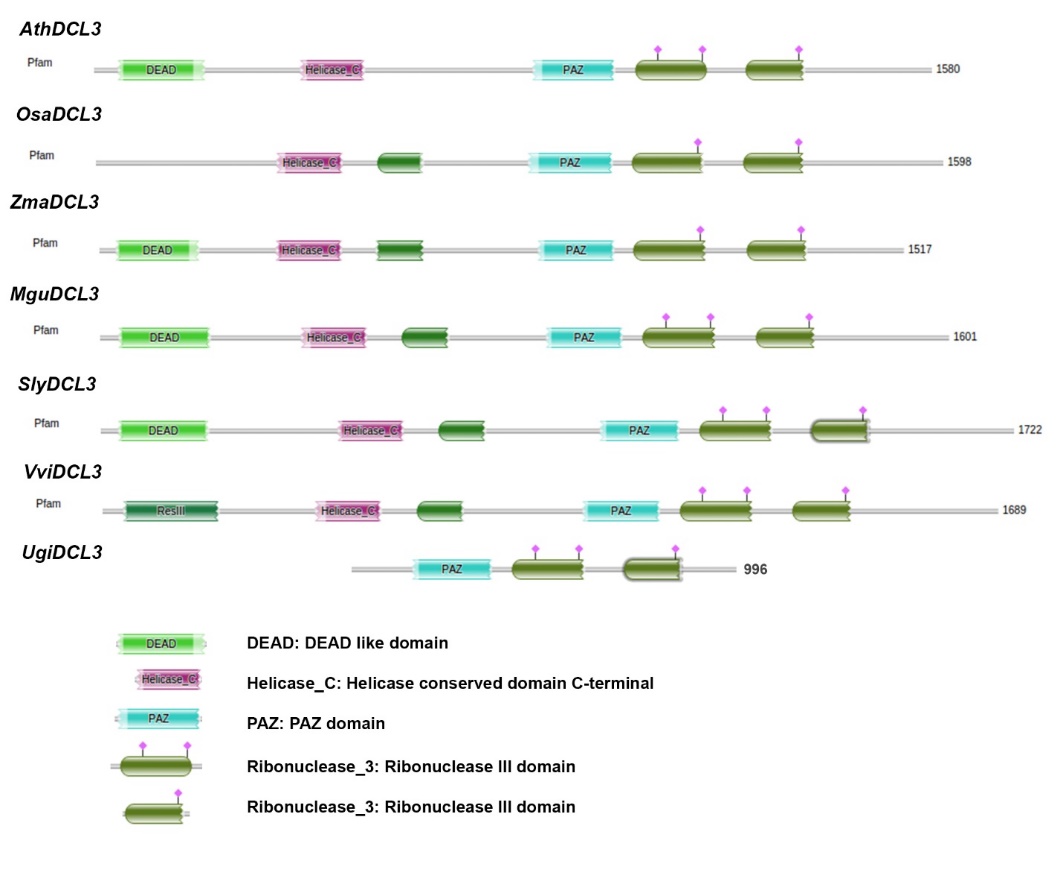


**Fig. S15. Conserved protein domains among DCL3 for some angiosperms**. Identification of conserved protein domains in different DCL3 proteins in plants in HMMER database (<http://hmmer.org/>).

^
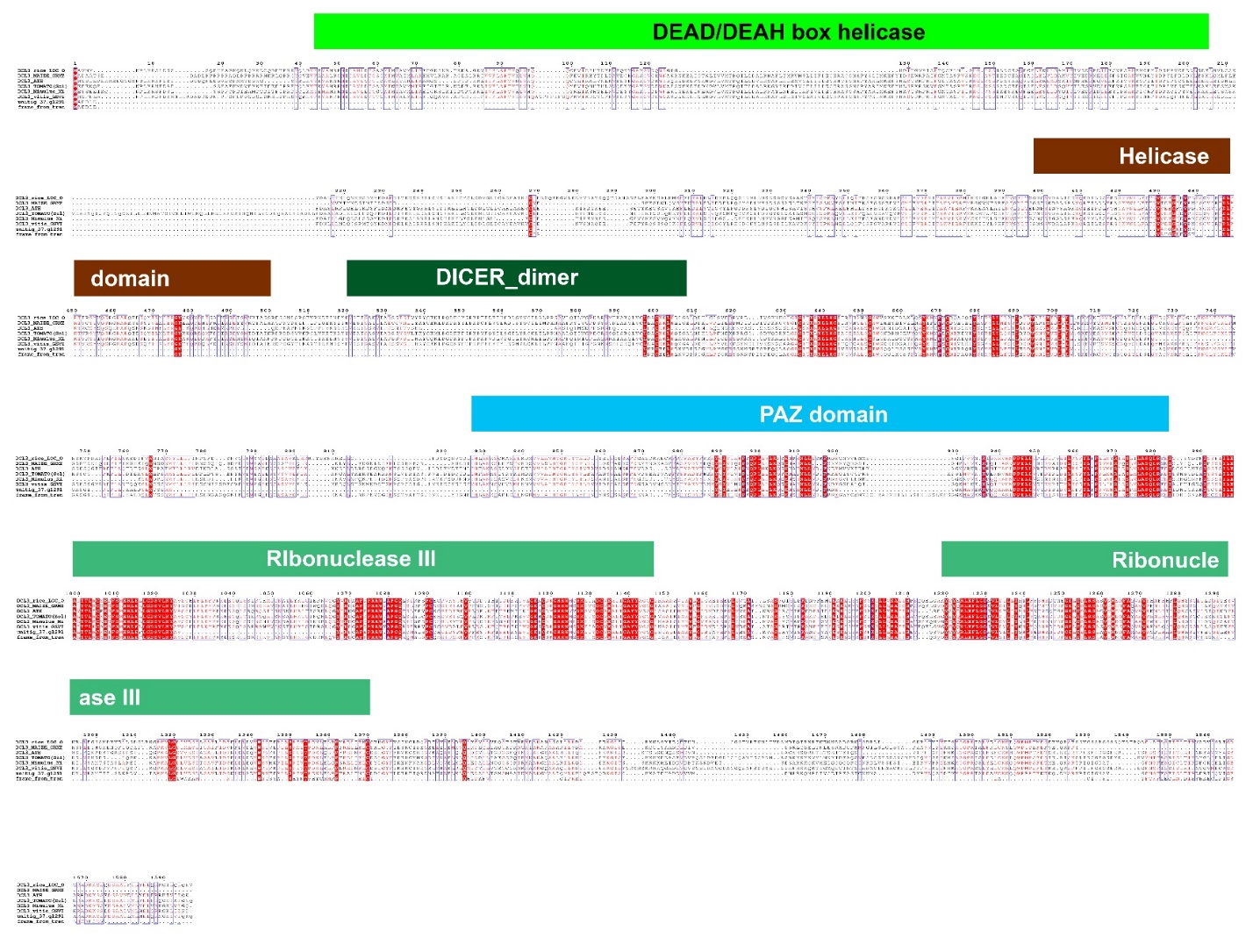
^

**Fig. S16. Multiple Sequence Alignment of some DCL3 proteins**. A multiple sequence alignment where red block represented the conserved protein domains with a representation in color blocks the typical protein domains for DCL3.


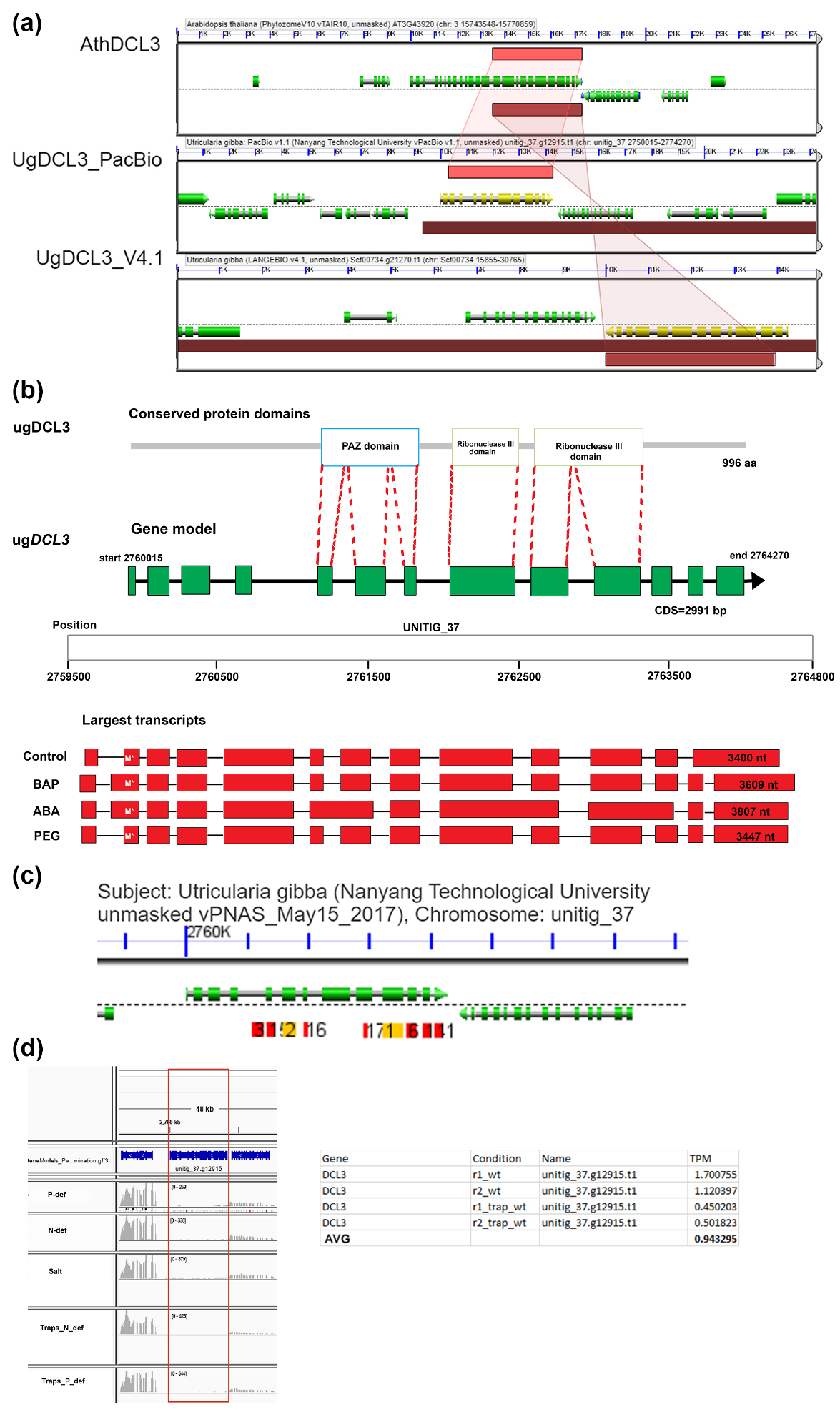


**Fig. S17. Ug*DCL3* in the transcriptomic datasets and RACE-PCR. (a)** Synteny analysis between *U. gibba* two gene models (two latest genome version) and A. thaliana DCL3, the colored blocks represent the syntenic regions. **(b)** The protein of ugDCL3 comprises 996 aminoacids (aa) and has three conserved protein domains (PAZ domain, and two contiguous Ribonuclease III domains). The gene model has a coding sequence of size 2991 bp and contains 13 exons which can be visualize in the protein conserved domains. The ug*DCL3* is located on the unitig_37 one of the largest contig. In the bottom we show the search of largest transcripts in our transcriptomic data of contrasting conditions. The conditions with largest transcripts were Control (Normal conditions), BAP (treatment with the hormone Benzil-aminopurine), ABA (treatment with Abscisic acid plant hormone) and PEG (Polyethylene glycol, an osmotic treatment). The transcript size varies among these conditions, but the coding sequence is the same. **(c)** 5´Race-PCR sequence alignment against reference genome highlighting in red blocks the 5´RACE-PCR and in green blocks de CDS sequence of *DCL3* gene (**d**) left: IGV browser location of *DCL3* gene and compared with reads coverage of some RNA-Seq libraries. Right: Table of transcripts per million (TPM) in WT libraries in *DCL3* gene.


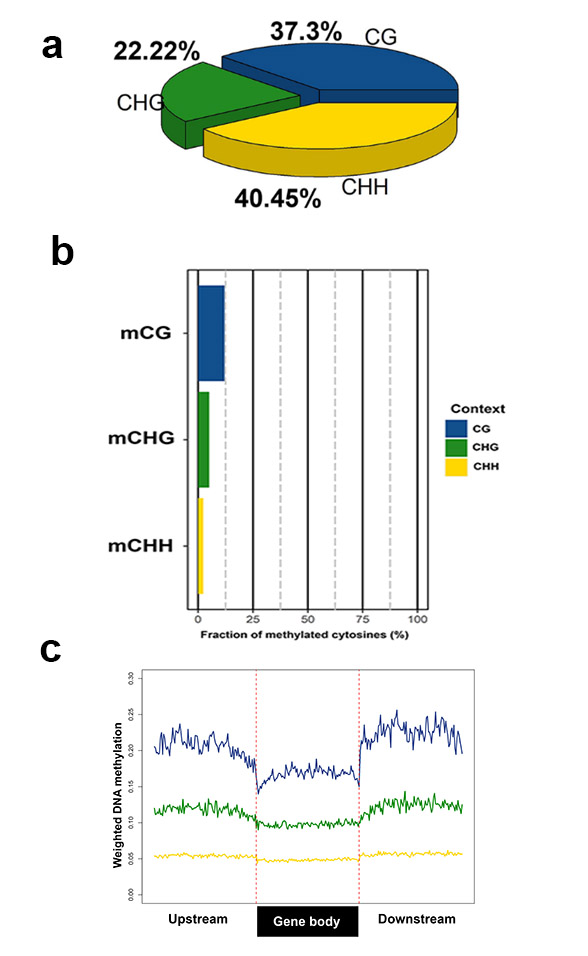


**Fig. S18. Methylation identification with SMRT-Seq. (a)** Pie plot representing global methylation context for m5Cs in the *U. gibba* genome. **(b)** Methylation levels for each methylation context. **(c)** Gene body methylation and 2 kp upstream and downstream the gene body.


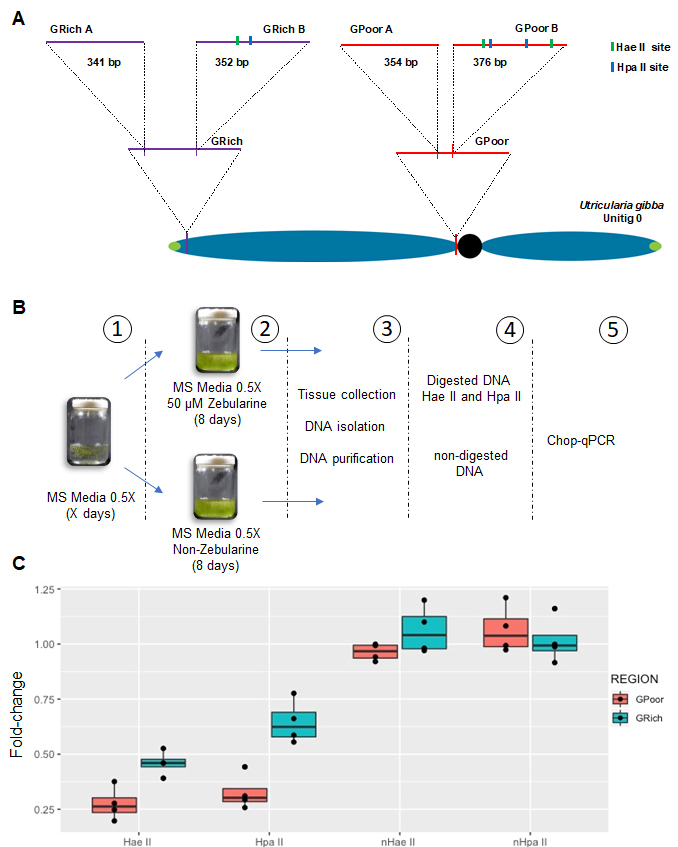


**Fig. S19. Experimental validation of Pac-Bio methylome data.** **(a)** Selection of genomic regions for Chop-qPCR Analysis. Two genomic regions (~ 9 Kb each) were selected from Utricularia gibba Unitig 0 for analysis, one gene rich region (GRich, purple line) and one gene poor region (GPoor, red line (pericentromeric)), within each of these particular regions two independent regions with similar DNA methylation density values presenting restriction sites for either Hpa II (green line) and Hae II (blue line) (GRich B, GPoor B) DNA methylation sensitive enzymes or absence of the same restriction sites (GRichA, GRichB) were selected. **(b)** Schematic representation of the Chop-qPCR experiment workflow. Vegetative tissue explants fom *Utricularia gibba* plants grown in MS media 0.5 X (MS 0.5X) for X days (1), were transferred to new MS 0.5X recipients containing either 0 (non-Zeb treatment) or 50 M Zebularine (Zeb treatment) for 8 days (2). Tissue of both Zeb and non-Zeb treatment was collected for genomic DNA isolation and purification (3). Purified Utricularia gibba genomic DNA was either non-digested or digested with methylation sensitive restriction enzymes Hae II or Hpa II (restriction is decreased or blocked if there is 5mC methylation inside recognition sequence) (4). Chop-qPCR was performed using digested and nondigested DNA from Zeb and non-Zeb samples (5). **(c)** Chop-qPCR of *Utricularia gibba* Unitig 0 GRich and Gpoor regions. Average of the amplification values of Unitig 0 regions without Hae II and Hpa II sites were used as internal controls (GRich A, GPoor A); non-Zebularine treatment values for each time point were used as calibrator samples. Values are foldchange of either digested (Hae II, Hpa II) and non-digested (non-Hae II, non-Hpa II) Zebularine treatments compared with non-Zebularine treatments, eg. a fold-change value lower than 1 indicates a decrease in the amplification of a fragment in the Zebularine treatment. Data presented are means of two biological replicates ± SEM (of two technical replicates each).


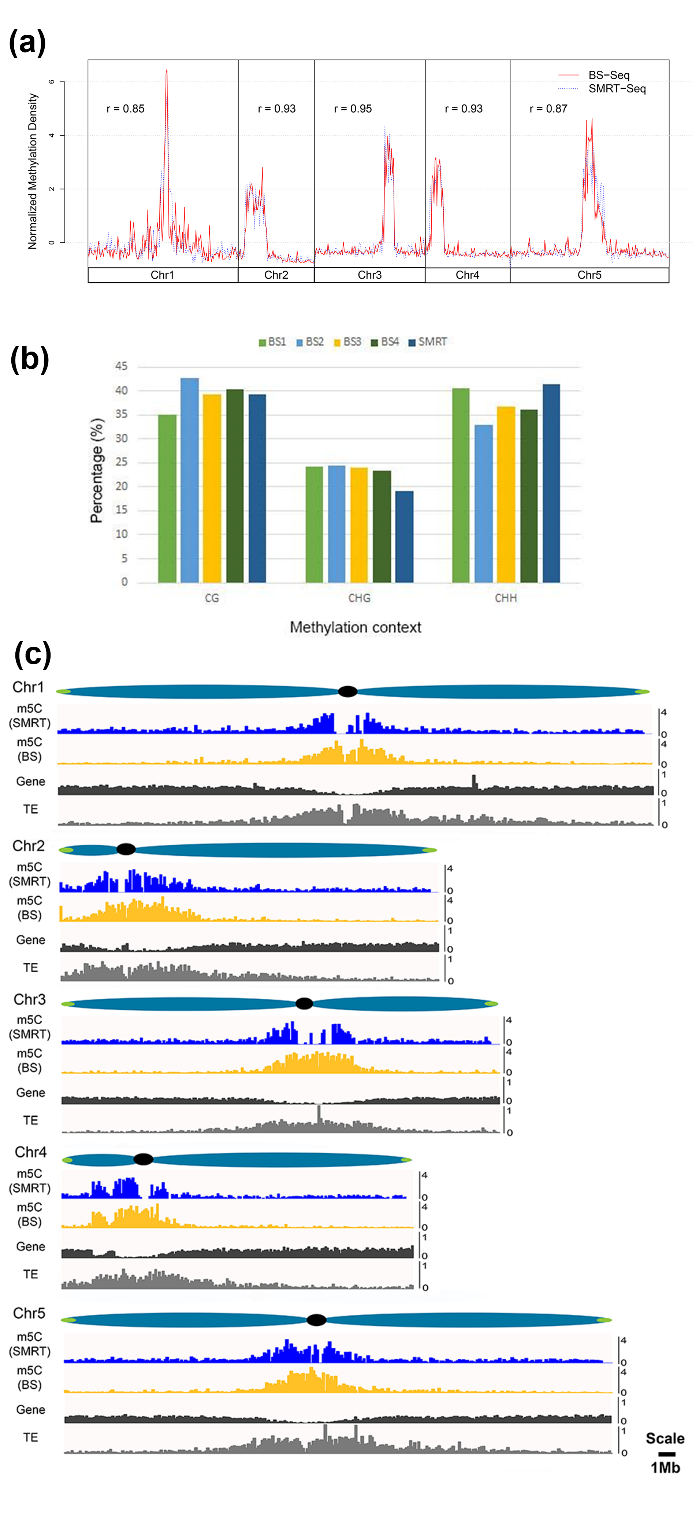


**Fig. S20. Comparison between m5C identification in *A. thaliana* with BS-Seq and SMRT-Seq. (a)** Distribution of m5C in the Arabidopsis genome in syntenic regions. Normalized methylation distribution estimated by BS-Seq and SMRT-Seq. In the histogram were represented 604 syntenic windows at genome-wide level divided by chromosome. The red line represents the density distribution of BS-Seq, and blue line represents the SMRT-Seq density. The estimated rˆ is given in purple for each chromosome. Y-axis is the normalized density methylation for pearson´s correlation. **(b)** Global methylation context for total m5C identification in 4 libraries of BS-Seq and SMRT-Seq. The figure shows the methylation context for individual BS-Seq libraries and SMRT-Seq of Arabidopsis. For BS-Seq were used 4 libraries named B1 (shoot 7days), B2 (Root 7 days), B3 (Shoot 16 days), B4 (Root 16 days) grown in optimal conditions and SMRT. In the X axis can see the methylation context (CG, CHG, CHH) grouped for different libraries. The Y axis represents the percentage for fraction of methylated cytosines in each context. **(c)** Global m5C distribution at chromosome scale. Y-axis shown the m5C density distribution in Z-score.

**Table S1. lncRNAs features. Summary of lncRNAs size and exon number.**

| **Mean**  **Size (nt)** | **Median Size (nt)** | **Largest**  **Size (nt)** | **Median exon number** | **Exon number** | **Number**  **lncRNAs** | **Mean**  **Size (nt)** |
| --- | --- | --- | --- | --- | --- | --- |
| 339.96 | 294 | 2134 | 1 | 1 | 3716 | 329.8652 |
|  |  |  |  | 2 | 482 | 380.4295 |
|  |  |  |  | 3 | 72 | 502.6944 |
|  |  |  |  | 4 or more | 25 | 592.4 |

**Table S2. List of microRNAs identified by Shortstack.**

| **Region** | **Strand** | **Sequence** | **Size** |
| --- | --- | --- | --- |
| unitig_0:1071117-1071283 | - | AAGCUCAGGAGGGAUAGCGCC | 21 |
| unitig_0:3967533-3967612 | + | CGAAUCUUGAUGAUGCUGCAU | 21 |
| unitig_0:6526104-6526300 | - | UUGGACUGAAGGGAGCUCCCU | 21 |
| unitig_0:7678166-7678348 | - | UGACAGAAGAGAGUGGGCAU | 20 |
| unitig_0:7685615-7685719 | - | GCUCUCUAUCUUUCUGUCACC | 21 |
| unitig_0:8065549-8065640 | + | UGACAGAAGAGAGUGAGCAC | 20 |
| unitig_8:1381776-1381866 | + | UCGGACCAGGCUUCAUUCCCC | 21 |
| unitig_8:5189790-5189891 | + | UCGGACCAGGCUUCAUUCCUC | 21 |
| unitig_22:573458-573562 | + | CCGGCGUCGUCAUUGCACCAC | 21 |
| unitig_22:831214-831512 | + | UCUCCAAACCGUUGUGCAGCC | 21 |
| unitig_22:1263224-1263458 | - | CUGAAGUGUUUGGGGUAACUC | 21 |
| unitig_22:3924210-3924398 | - | UCGGACCAGGCUUCAUUCCUC | 21 |
| unitig_22:5269543-5269675 | - | CAUGUGCCCAUCUUCCCCAUC | 21 |
| unitig_22:5853562-5853730 | + | UCGGACCAGGCUUCAUUCCCC | 21 |
| unitig_748:597466-597718 | - | UGACAGAAGAGAGUGAGCAC | 20 |
| unitig_748:2182336-2182455 | - | UGAUUGAGCCGUGCCAAUAUC | 21 |
| unitig_748:3972706-3972838 | + | UCGCUUGGUGCAGGUCGGGAA | 21 |
| unitig_748:4270228-4270348 | - | GCUUACUCUCUAUCUGUCACC | 21 |
| unitig_748:4510927-4511135 | - | UGAAGCUGCCAGCAUGAUCUA | 21 |
| unitig_26:1007561-1007686 | + | CAGCCAAGGAUGAUUUGCCGG | 21 |
| unitig_26:1585944-1586182 | - | GUGCGAUUCUCCUUUGGCAUG | 21 |
| unitig_26:1587027-1587110 | + | UGCCAAAGGAGAUUUGCCCCA | 21 |
| unitig_26:4573583-4573657 | + | CGCGCAUAGCCUGGCACCAUG | 21 |
| unitig_32:433547-433621 | + | UGAAGCUGCCAGCAUGAUCUA | 21 |
| unitig_32:752373-752536 | - | UCGCUUGGUGCAGGUCGGGAA | 21 |
| unitig_32:1673744-1673877 | + | UGAUUGAGCCGUGCCAAUAUC | 21 |
| unitig_37:824050-824219 | + | UUGGACUGAAGGGAGCUCCCU | 21 |
| unitig_37:1020988-1021074 | - | UGCCUGGCUCCCUGUAUGCCA | 21 |
| unitig_749:538338-538398 | + | AGGUGCCACGCUGUGUGCGUC | 21 |
| unitig_749:2563622-2563769 | + | AAGCUCAAGAGGGAUAGCGCC | 21 |
| unitig_749:2889882-2890016 | + | CAGCCAAGGAUGAUUUGCCGG | 21 |
| unitig_899:215905-216007 | - | UGCCUGGCUCCCUGUAUGCCA | 21 |
| unitig_899:1079430-1079588 | - | CACGUGCUCCCUUCUCCACC | 20 |
| unitig_899:1411463-1411537 | + | UGAAGCUGCCAGCAUGAUCUU | 21 |
| unitig_899:2231668-2231851 | + | UCUCGGACCAGGCUUCAUUCC | 21 |
| unitig_899:2895922-2896015 | + | UUCCACAGCUUUCUUGAACUU | 21 |
| unitig_899:2898407-2898540 | + | UGACAGAAGAGAGUGAGCGC | 20 |
| unitig_899:2906016-2906129 | + | UGACAGAAGAGAGUGAGCGC | 20 |
| unitig_899:2907640-2907770 | + | UGACAGAAGAGAGUGAGCGCC | 21 |
| unitig_578:1613009-1613230 | + | CAGCCAAGGAUGACUUGCCGG | 21 |
| unitig_578:2399579-2399669 | + | UCGGACCAGGCUUCAUUCCUC | 21 |
| unitig_578:2850911-2851062 | + | UCGGACCAGGCUUCAUUCCCC | 21 |
| unitig_52:2405905-2405994 | - | UUUUCCGAUCCCUCCCAUUCC | 21 |
| unitig_52:2498299-2498406 | + | UGCACUGCCUCUUCCCUGGCUC | 22 |
| unitig_52:2812513-2812689 | + | UGACAGAAGAGAGCGAGCAC | 20 |
| unitig_52:2897996-2898206 | - | UUCUCCGGCGUCGAUUAUCUGA | 22 |
| unitig_46:610298-610418 | + | UGAUUGAGCCGUGCCAAUAUC | 21 |
| unitig_46:2516815-2517021 | + | UUGGACUGAAGGGAGCUCCCU | 21 |
| unitig_46:2649439-2649603 | - | UGCCUGGCUCCCUGUAUGCCA | 21 |
| unitig_46:3039631-3039920 | + | UUGGACUGAAGGGAGCUCCUU | 21 |
| unitig_46:3102029-3102112 | - | UGCCAAAGGAGAGUUGCCCUG | 21 |
| unitig_699:1182319-1182553 | + | UGGAGAAGCAGGGCACGUGCA | 21 |
| unitig_699:1600811-1601063 | + | CAGCCAAGGAUGACUUGCCGG | 21 |
| unitig_699:2616843-2616930 | + | ACACCACAUCAUUACAUUGGG | 21 |
| unitig_21:887515-887619 | + | UUCCACAGCUUUCUUGAACUU | 21 |
| unitig_21:1141952-1142171 | - | UUUGGAUUGAAGGGAGCUCUA | 21 |
| unitig_747:1099561-1099710 | - | UGAUUGAGCCGUGCCAAUAUC | 21 |
| unitig_747:2210919-2211042 | - | UCGCUUGGUGCAGGUCGGGAA | 21 |
| unitig_736:668718-668807 | + | AGAAUCUUGAUGAUGCUGCAU | 21 |
| unitig_736:791977-792080 | - | UGAAGCUGCCAGCUUGAUCUU | 21 |
| unitig_736:985139-985228 | - | UGCCUGGCUCCCUGUAUGCCA | 21 |
| unitig_736:1101798-1101997 | + | CUGACAGAAGAUAGAGAGCAC | 21 |
| unitig_736:1102064-1102305 | + | UUGACAGAAGAGAGAGGGCAC | 21 |
| unitig_744:1732110-1732225 | + | UUUGGAUUGAAGGGAGCUCUA | 21 |
| unitig_744:1863287-1863389 | - | UUCCACAGCUUUCUUGAACUU | 21 |
| unitig_744:2193635-2193746 | - | UGACAGAAGAGGGAGGGCACU | 21 |
| unitig_27:628456-628617 | - | CAUGUGCCCUUCUUCCCCACC | 21 |
| unitig_746:126348-126537 | + | UUCGCAGGAGAGACGGCACGCCG | 23 |
| unitig_746:721235-721432 | + | UGAUUGAGCCGCGCCAAUAUC | 21 |
| unitig_77:2256-2390 | - | CAGCCAAGGAUGAUUUGCCGG | 21 |
| unitig_45:19980-20057 | - | UCGGACCAGGCUUCAUUCCCC | 21 |

**Table S3. Canonical genes involved in miRNAs biogenesis.**


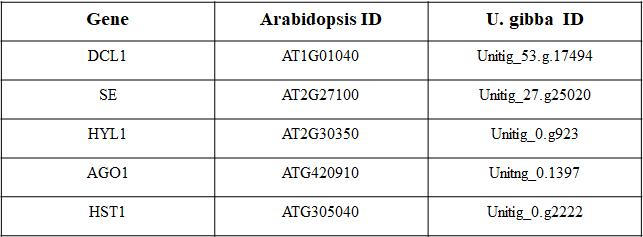


**Table S4. Ectopic gametic precursors and female gametophytes in the ovule of *Utricularia gibba*. ________________________________________________________________________________________**

                             Ovary size (mm)

0.3 – 0.5^a^ 0.5 – 1^a^

___________________________________________________________________________

One gametic precursor 51.4% (73)

Several gametic precursors 42.9% (61)

One female gametophyte 40.7% (31)

One female gametophyte

+ ectopic gametic precursors 22.4% (17)

Two female gametophytes 23.7% (18)

Degenerated female gametophyte 13.1% (10)

ND^b^ 5.7% (8)

Total 100% (142) 100% (76)

**_____________________________________________________________________________**

^a^ Brackets indicate the number of ovules observed with a given phenotype.

^b^ Not determined
